# Evolution and Taxonomic Classification of Human Papillomavirus 16 (HPV16)-Related Variant Genomes: HPV31, HPV33, HPV35, HPV52, HPV58 and HPV67

**DOI:** 10.1371/journal.pone.0020183

**Published:** 2011-05-27

**Authors:** Zigui Chen, Mark Schiffman, Rolando Herrero, Rob DeSalle, Kathryn Anastos, Michel Segondy, Vikrant V. Sahasrabuddhe, Patti E. Gravitt, Ann W. Hsing, Robert D. Burk

**Affiliations:** 1 Department of Microbiology and Immunology, Albert Einstein College of Medicine, Bronx, New York, United States of America; 2 Division of Cancer Epidemiology and Genetics, National Cancer Institute, Bethesda, Maryland, United States of America; 3 Proyecto Epidemiologica Guanacaste, Fundación INCIENSA, San José, Costa Rica; 4 Sackler Institute of Comparative Genomics, American Museum of Natural History, Bronx, New York, United States of America; 5 Department of Medicine, Montefiore Medical Center, Bronx, New York, United States of America; 6 Department of Biology and Pathology, Montpellier University Hospital, Montpellier, France; 7 Institute for Global Health, Vanderbilt University, Nashville, Tennessee, United States of America; 8 Department of Epidemiology, Johns Hopkins Bloomberg School of Public Health, Baltimore, Maryland, United States of America; 9 Department of Epidemiology and Population Health, Albert Einstein College of Medicine, Bronx, New York, United States of America; 10 Department of Obstetrics, Gynecology and Women's Health, Albert Einstein College of Medicine, Bronx, New York, United States of America; 11 Department of Pediatrics, Albert Einstein College of Medicine, Bronx, New York, United States of America; Tsan Yuk Hospital, Hospital Authority, China

## Abstract

**Background:**

*Human papillomavirus 16* (HPV16) species group (alpha-9) of the *Alphapapillomavirus* genus contains HPV16, HPV31, HPV33, HPV35, HPV52, HPV58 and HPV67. These HPVs account for 75% of invasive cervical cancers worldwide. Viral variants of these HPVs differ in evolutionary history and pathogenicity. Moreover, a comprehensive nomenclature system for HPV variants is lacking, limiting comparisons between studies.

**Methods:**

DNA from cervical samples previously characterized for HPV type were obtained from multiple geographic regions to screen for novel variants. The complete 8 kb genomes of 120 variants representing the major and minor lineages of the HPV16-related alpha-9 HPV types were sequenced to capture maximum viral heterogeneity. Viral evolution was characterized by constructing phylogenic trees based on complete genomes using multiple algorithms. Maximal and viral region specific divergence was calculated by global and pairwise alignments. Variant lineages were classified and named using an alphanumeric system; the prototype genome was assigned to the A lineage for all types.

**Results:**

The range of genome-genome sequence heterogeneity varied from 0.6% for HPV35 to 2.2% for HPV52 and included 1.4% for HPV31, 1.1% for HPV33, 1.7% for HPV58 and 1.1% for HPV67. Nucleotide differences of approximately 1.0% - 10.0% and 0.5%–1.0% of the complete genomes were used to define variant lineages and sublineages, respectively. Each gene/region differs in sequence diversity, from most variable to least variable: noncoding region 1 (NCR1) /noncoding region 2 (NCR2) >upstream regulatory region (URR)> E6/E7 > E2/L2 > E1/L1.

**Conclusions:**

These data define maximum viral genomic heterogeneity of HPV16-related alpha-9 HPV variants. The proposed nomenclature system facilitates the comparison of variants across epidemiological studies. Sequence diversity and phylogenies of this clinically important group of HPVs provides the basis for further studies of discrete viral evolution, epidemiology, pathogenesis and preventative/therapeutic interventions.

## Introduction

Persistent infection of specific types of genital human papillomaviruses (HPVs) is the central cause of cervical cancer and its precursor, cervical intraepithelial neoplasia (CIN). Cervical cancer is the most common gynecologic malignancy and one of the leading causes of cancer mortality in women worldwide [1], [2]. Over 150 HPV types have been fully characterized; approximately sixty of these are predominantly detected in the cervical epithelia and sort to the *Alphapapillomavirus* genus [3], [4]. Most oncogenic or high-risk (HR) types associated with invasive cervical cancer[5], [6], [7] are phylogenetically clustered within either the *Human papillomavirus 16* (alpha-9) or *Human papillomavirus 18* (alpha-7) species groups [8], and account for ∼75% and ∼15% of all cervical cancers worldwide, respectively [6], [7].

Human papillomavirus nomenclature is established by the International Committee on Taxonomy of Viruses (ICTV) based on recommendations from the Study Group of Papillomavirus [3], [4], [9]. The ICTV uses strict definitions for genera and species, but does not set standards below the species level [10]. Papillomavirus researchers evolved a “community” nomenclature that has been widely embraced and extremely useful in epidemiological studies [3], [4]. A distinct papillomavirus (PV) “type” is established when the nucleotide sequence of the L1 gene of a cloned virus differs from that of any other characterized types by at least 10% [3], [4]. To date, the ICTV has not recognized the “type” terminology, nor the naming of species group by number [9]. In addition, the lexicon of lower taxonomic levels, such as serotypes, strains, variants are not under the aegis of the ICTV. Within the PV research community, isolates of the same HPV type are referred to as variants or subtypes when the nucleotide sequences of their L1 genes differ by less than 10%. Except for HPV16, HPV18, HPV45 and HPV97 there has not been a systematic study of HPV genome variation, nor a logical and standard classification system of variant lineages [11], [12]. Given that the HPV alpha-9 group plays such an important role in human cancer and variant lineages have different pathologic potentials, a comprehensive evolutionary study and classification system is needed. For instance, the upstream regulatory region (URR) sequences have most often been used to describe intratypic diversity and variant lineages. However, it would be valuable to relate changes throughout the genome with specific variant lineages.

HPV16 and HPV18 partial and complete viral sequences form evolutionary trees with the bifurcation driven by variants with high prevalences in cohorts from different regions of the world [13], [14]. This evolutionary divergence is reflected in the phylogeny of these strains and is reminiscent of the migration patterns of *Homo sapiens* and suggests that HPV variant lineages may have co-diversified with human populations as they exponentially expanded across the planet. The intratypic evolutionary studies of HPV16 and HPV18 variants were initially inferred from the partial URR and E6 sequences, and have been recently expanded to include the complete genomes [11], [12], [15]. Subsequent studies have investigated the genetic variation of other HPV types. Comparisons of isolates of HPV6 and 11 [16], [17], HPV5 and 8 [18], [19], HPV2, 27 and 57 [20], HPV44 and 68 [21], HPV53, 56 and 66 [22], HPV31, 33, 35, 52 and 58 [23], [24], [25], [26], [27], [28], and other rare HPV types [29] have confirmed that each type demonstrates various degrees of genomic diversity, although the association of variant lineages and geographic origins remains a bit murky, probably due to sampling biases. However, most previous studies have sampled small, partial regions of the viral genome generally limited to the E6 and L1 open reading frames (ORFs) and the URR region. A systematic study of HPV variants and the evolutionary dynamics has not been established for the HPV16-related alpha-9 types.

Despite phylogenetic relatedness, HPV variants can differ in pathogenicity. There is a three-fold or greater risk of cervical cancer for Asian-American (AA) or African (Af) HPV16 variants compared to European (E) variants; and, non-European variants of HPV18 may be more common in cancer tissues and high-grade cervical lesions [30], [31], [32]. HPV16 and HPV18 variants confer different risks of viral persistence and/or progression to precancer/cancer. Nevertheless, there is almost no data on the natural history of other high-risk (HR) HPV variants. For example, it is not known whether HPV31, a less-studied oncogenic type, represents a homogeneous or heterogeneous set of variants with similarities or differences in viral persistence and/or oncogenicity. Although an HPV33 variant (C7732G) and an HPV58 variant (C632T and G760A) have been reported to be associated with a higher risk of cervical cancer [33], [34], the lack of a coherent classification and nomenclature system for HPV variant lineages does not facilitate comparison with other studies that measure variability in a different region of the viral genome for classification. Moreover, specific variants occur on lineages fixed by stochastic processes that likely include some type of, as yet unmeasured, natural selection for increased viral fitness [11], [35].

In this report, the complete 8 kb genomes of 120 variants representing major lineages and sublineages of HPV16-related alpha-9 types (HPV31, 33, 35, 52, 58 and 67) were selected and sequenced to capture maximum viral heterogeneity. Variations across the genomes were identified and the evolutionary phylogeny and nomenclature of the alpha-9 variant lineages are described and will enable future studies of their discrete evolution, epidemiology, pathogenicity and vaccine response differences.

## Materials and Methods

### Clinical specimens, identification of novel HPV variants and whole genome sequencing

DNA from cervicovaginal samples already determined to have HPV16-related alpha-9 types (HPV31, 33, 35, 52, 58 and 67) by previous testing were available from women participating in epidemiological studies worldwide, including - Costa Rica [36], Taiwan [37], Thailand [38],[39], Rwanda [40], Burkina Faso [41] and Zambia [42]. The methods for sample collection and HPV typing are provided in the references from each study. The number of samples analyzed for each type is shown in Table 1. The HPV genomes within the DNA samples were classified by sequencing the URR and/or E6 regions from PCR products as described [43]. Briefly, we used type-specific primers to amplify a partial fragment of the URR region and/or the E6 ORF using a one-tube nested PCR method [44]. The E6 ORF was evaluated only for those specimens that did not yield data for the URR region. The PCR product sizes were confirmed by gel electrophoresis, purified using the QuickStep 2 PCR Purification kit (Edge BioSystems, Gaithersburg, MD) or QIAquick Gel Extraction kit (Qiagen, Valencia, CA) and submitted for sequencing of both strands at the Einstein Genomics Facility. The sequences for each type under study were separately aligned and preliminary phylogenetic trees were used to identify samples that likely contained diverse viral genomes (data not shown). Based on this analysis, we selected type-specific viral isolates for complete genome sequencing that (1) represented different variant clades or (2) had 2 or more isolates that contained at least 2 unique sequence variations (e.g., single nucleotide polymorphisms (SNPs)) not present in other isolates within the URR/E6 regions.

**Table 1:** Summary of HPV isolates and sequenced complete genomes.

HPV type	Total no. sampled a	Total no. sequenced b	Genome size (nucleotides) c	Variable nucleotide positions d	Total potential amino acid positions e	Variable amino acid positions f
31	316	22	7967 (7878, 7945)	299 (3.8%)	2302	109 (4.7%)
33	179	20	7912 (7830, 7912)	186 (2.4%)	2284	69 (3.0%)
35	214	23	7908 (7876, 7908)	140 (1.8%)	2306	46 (2.0%)
52	481	22	7993 (7933, 7974)	354 (4.4%)	2326	105 (4.5%)
58	447	26	7837 (7814, 7836)	400 (5.1%)	2326	148 (6.4%)
67	32	7	7825 (7801, 7819)	135 (1.7%)	2321	45 (1.9%)

a Number of HPV isolates characterized by sequencing the URR ± E6 region;

b Number of isolate genomes sequenced;

c Number of nucleotide sequences within the genome based on one genome size for each HPV type calculated from the global sequence alignments (see Materials and Methods). Minimum and maximum lengths of sequenced genomes for each type are shown and represent the presence of insertion and deletions (indels);

d Total number and percentage of variable nucleotide positions based on one genome size for each HPV type as described above. Nucleotide variations include single nucleotide polymorphisms (SNPs) and indels, which are considered equivalent to one variation per indel independent of indel size;

e Maximum number of encoded amino acids (not including overlapping ORFs) based on one genome size for each type as described above. Cumulative number of amino acids are taken from 7 ORFs (E6, E7, E1, E2, E5, L2 and L1), E4 is not counted separately nor are other overlapping ORFs;

f Total number and percentage of variable amino acids based on the total number of amino acid positions derived from the established genome size for each HPV type.

The subset of viral genomes sequenced captured the maximum diversity noted in sequencing the URR/E6 regions. The number of genomes selected for sequencing for each type was based on identification of divergent isolates and differed for each type (table 1). The complete 8 kb genomes from clinical samples were amplified in 2 to 3 overlapping fragments using type-specific primer sets (available from authors) based on the prototype sequence of each type. For overlapping PCR, an equal mixture of AmpliTaq Gold DNA polymerase (Applied Biosystems, Carlsbad, CA) and Platinum Taq DNA Polymerase (Invitrogen, Carlsbad, CA) were utilized as previously described [11], [12], [45]. PCR products of anticipated size, as determined by gel analyses, were either directly sequenced or cloned into pGEM-T easy (Promega, Madison, WI) or TOPO TA pCR2.1 vectors (Invitrogen, Carlsbad, CA) and then sequenced. Comparison of repeat sequencing of PCR products from the same isolates resulted in a difference of less than one change per 8,000 bp; whereas, comparison of the cloned genomes gave a difference of approximately one difference per 5,000 bp. For discrepancies between sequences, we used the sequence of the PCR product as the valid sequence. HPV complete genome sequences were submitted to GenBank; the accession numbers are listed in Table S1.

### Evolutionary analyses and phylogenetic tree construction

The nucleotide sequences of the complete circular genomes were linearized at the first ATG of the E1 ORF and globally aligned using the program MAFFT v6.846 [46]. Based on the concept of a single ancestor for each type, a unique genome size is assigned to each HPV type based on the global alignment and the variation in genome size of the isolated variants is ascribed to insertions and deletions (indels). Each indel was counted as one event. The assignment of position numbers for each nucleotide is based on the nucleotide numbering of the prototype reference sequence.

**Figure 1:**
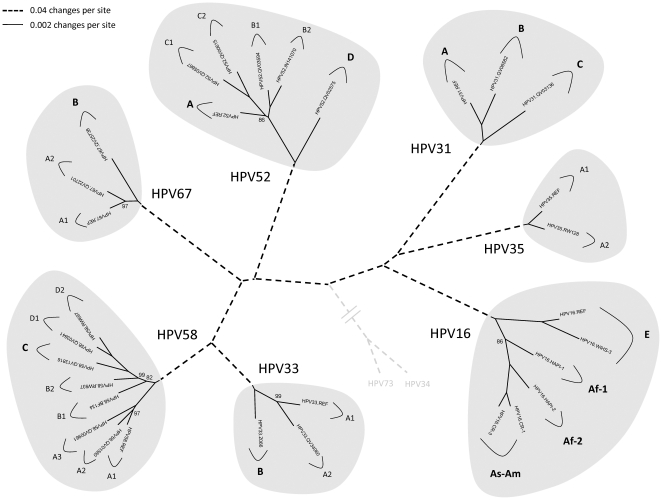
Alpha-9 phylogenetic tree showing representative types and variant lineages. A phylogenetic tree was constructed using the MrBayes (v3.1.2) program [48] inferred from the global alignment of complete circular genome nucleotide sequences linearized at the first ATG of the E1 ORF. To root the tree, HPV34 and HPV73 prototype sequences (NCBI accession numbers NC_001587 and NC_006165, respectively) were set as the outgroup and are represented by grey broken lines. The Bayesian credibility values less than 100 were indicated on or near the branch nodes. The shaded areas represent groupings of lineages and sublineages of HPV16, HPV31, HPV33, HPV35, HPV52, HPV58 and HPV67. The length of broken and solid lines represent distance between clades, although the number of changes is different for these two lines, the scale is indicated in the upper left corner of the figure.

MrBayes v3.1.2 [47], [48] with 10,000,000 cycles for the Markov chain Monte Carlo (MCMC) algorithm was used to generate phylogenetic trees from the aligned complete genome nucleotide sequences. For Bayesian tree construction, the computer program ModelTest v3.7 [49] was used to identify the best evolutionary model; the identified gamma model was set for among-site rate variation that allowed substitution rates of different sites to vary. Maximum parsimony (MP) and neighbor joining (NJ) trees were calculated by a heuristic search in PAUP* v4.0b10 [50]. For maximum parsimony analyses, nucleotide sequences were reduced to phylogenetically informative sites. Data were bootstrap resampled 1,000 times. Trees are shown in Figures 1 – [Fig pone-0020183-g002] [Fig pone-0020183-g003] [Fig pone-0020183-g004] [Fig pone-0020183-g005] [Fig pone-0020183-g006] 7.

**Figure 2 pone-0020183-g002:**
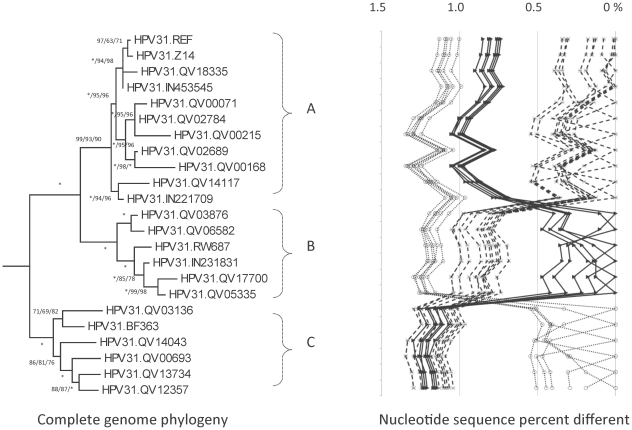
HPV31variant tree topologies andpairwise comparisons of individual complete genomes. Bayesian trees were inferred from global alignment of complete genome nucleotide sequences (the other HPV16-related HPV reference prototypes were set as the outgroup). Numbers on or near branches indicate support indices in the following order: Bayesian credibility value using MrBayes v3.1.2 [48], maximum parsimony (MP) bootstrap percentage and neighbor joining (NJ) bootstrap percentage using PAUP* v4.0b10 [50]. An asterisk (*) indicates 100% agreement between methods. “NA” reflects disagreement between a method and the reference Bayesian tree at a given node. Thus, one tree is shown, but three different methods of tree construction were used to estimate the support of the provided tree, as explained above. Distinct variant lineages (i.e., termed A, B, and C) are classified according to the topology and nucleotide sequence differences from >1% to <10%. The percent nucleotide sequence differences were calculated for each isolate compared to all other isolates of the same type based on the complete genome nucleotide sequences and are shown in the panel to the right of each phylogeny. Values for each comparison of a given isolate are connected by lines and the comparison to self is indicated by the 0% difference point. Symbols and lines used are different for each distinct variant lineage to facilitate visual comparisons. For example, percentage differences of variant lineage A are indicated by X's and connected by broken lines; values for isolates of variant lineage B are indicated by closed triangles and connected by solid lines; and, difference values of lineage C isolates are indicated by open circles and connected by a dotted line.

**Figure 3 pone-0020183-g003:**
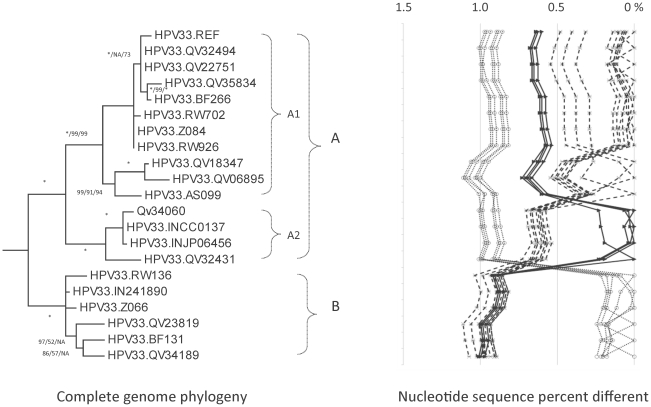
HPV33 variant tree topologies and pairwise comparisons of individual complete genomes. The phylogenetic tree was constructed as described in Figure 2. Distinct variant lineages (i.e., termed A and B) are classified according to the topology and nucleotide sequence differences from >1% to <10%. Distinct sublineages (i.e., termed A1 and A2) were also inferred from the tree topology and nucleotide sequence differences in the >0.5% to <1% range. The percent nucleotide sequence differences were calculated and are shown in the panel to the right of each phylogeny as described in Figure 2.

**Figure 4 pone-0020183-g004:**
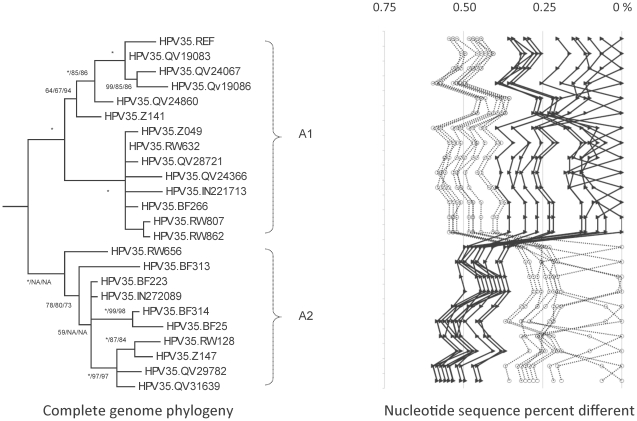
HPV35 variant tree topologies and pairwise comparisons of individual complete genomes. The phylogenetic tree was constructed as described in Figure 2. There were no distinct variant lineages however, sublineages (i.e., termed A1 and A2) were inferred from the tree topology and nucleotide sequence differences in the >0.5% to <1% range. The percent nucleotide sequence differences were calculated and are shown in the panel to the right of each phylogeny as described in Figure 2.

**Figure 5 pone-0020183-g005:**
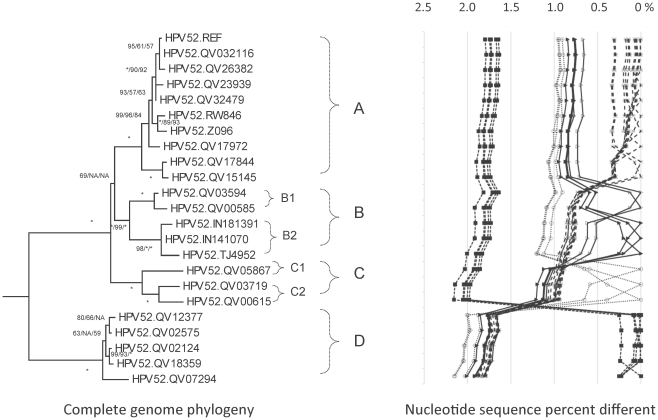
HPV52 variant tree topologies and pairwise comparisons of individual complete genomes. The phylogenetic tree was constructed as described in Figure 2. Distinct variant lineages (i.e., termed A, B, C and D) are classified according to the topology and nucleotide sequence differences from >1% to <10%. Distinct sublineages (i.e., termed B1, B2, C1 and C2) were also inferred from the tree topology and nucleotide sequence differences in the >0.5% to <1% range. The percent nucleotide sequence differences were calculated and are shown in the panel to the right of each phylogeny as described in Figure 2.

**Figure 6 pone-0020183-g006:**
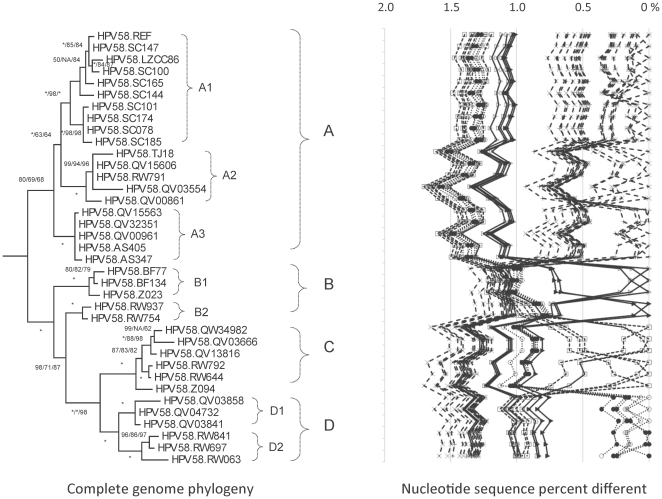
HPV58 variant tree topologies and pairwise comparisons of individual complete genomes. The phylogenetic tree was constructed as described in Figure 2. Distinct variant lineages (i.e., termed A, B, C and D) are classified according to the topology and nucleotide sequence differences from >1% to <10%. Distinct sublineages (i.e., termed A1, A2, A3, B1, B2, D1 and D2) were also inferred from the tree topology and nucleotide sequence differences in the >0.5% to <1% range. The percent nucleotide sequence differences were calculated and are shown in the panel to the right of each phylogeny as described in Figure 2.

**Figure 7:**
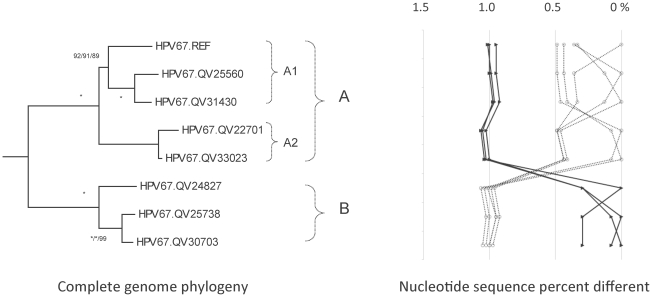
HPV67 variant tree topologies and pairwise comparisons of individual complete genomes. The phylogenetic tree was constructed as described in Figure 2. Distinct variant lineages (i.e., termed A and B) are classified according to the topology and nucleotide sequence differences from >1% to <10%. Distinct sublineages (i.e., termed A1 and A2) were also inferred from the tree topology and nucleotide sequence differences in the >0.5% to <1% range. The percent nucleotide sequence differences were calculated and are shown in the panel to the right of each phylogeny as described in Figure 2.

Separate Bayesian trees were inferred from nucleotide sequences of the “early genes” (E6, E7, E1, E2 and E5) and “late genes” (L2 and L1), in order to assess changes in tree topology [51]. The amino acids of each ORF were aligned using MUSCLE v3.7 [52] within the Seaview v4.1 program [53]; the nucleotide sequences of each codon region were then aligned using the corresponding aligned amino acid sequences.

**Figure 8:**
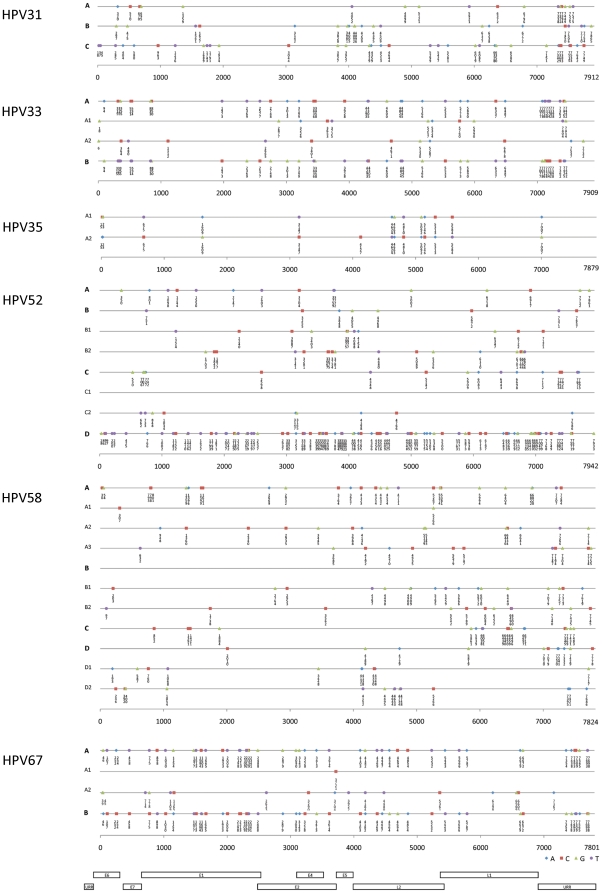
Diagnostic lineage-specific single nucleotide polymorphisms (SNPs) and their position in the genome. Lineage-specific SNPs were determined from alignments of type specific variants using the program MacClade v4.08 [55]. The position of variants across HPV lineage(s) and sublineage(s) are displayed to the right of the name of the clade from which the data was abstracted, as depicted in the phylogenetic trees in Figures 2–7. The viral genome sequence differences for each sequenced isolate are displayed in Figure S2. Regions of the genome are displayed below the *x*-axis for reference. The graphic output was generated using Microsoft Excel.

**Figure 9:**
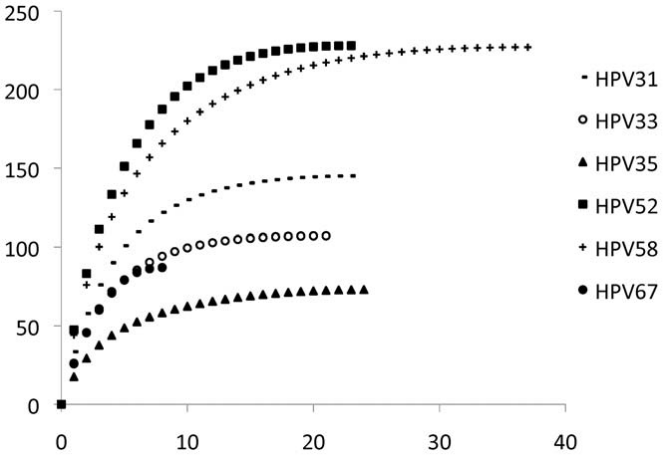
Single-nucleotide polymorphism (SNP) rarefaction curves. The program EstimateS v8.2.0 for Mac OS (downloaded from: http://viceroy.eeb.uconn.edu/EstimateS) was used to illustrate the curves. The Y-axis represents the total number of parsim-informative single nucleotide polymorphisms (SNPs) observed in at least 2 genomes of a specific type. Insertion and deletions are counted as one event equal to a single SNP. The X-axis shows the number of sequenced isolates. The curve generated for variants of each HPV type are displayed by different lines as indicated by the key to the right of the curves. For reference, the number of variable nucleotide positions for HPV31, HPV33, HPV35, HPV52, HPV58 and HPV67 genomes are 3.8%, 2.4%, 1.8%, 4.4%, 5.1% and 1.7%, respectively (see Table 1).

SNPs within the HPV genomes and lineage-specific SNPs were determined from alignments of type specific variant genomes using MEGA5 [54] and MacClade v4.08 [55], respectively and are displayed in Figure 8. Positions of SNPs and indels are based on the prototype reference sequence. Mean nucleotide differences and standard errors between and within type-specific lineages and sublineages were calculated from the global sequence alignment of each type using MEGA5 bootstrapped 1,000 times [54]. The rarefaction curves (shown in Figure 9) for each type were generated by EstimateS v8.2 [56].

## Results

Human papillomavirus isolates of known types were obtained from previously characterized cervicovaginal exfoliated cells from a variety of studies performed in different geographic locations. We reasoned that this large set of clinical materials would capture a major portion of the genomic diversity of the HPV16-related HPV types. The core set of clinical samples originated from a large (10,000 women) population-based study of HPV and cervix neoplasia in Costa Rica [43], and was supplemented with clinical materials obtained from 3 regions in Africa - Burkina Faso [41], Rwanda [40], and Zambia [42]; and two locations in Asia – Thailand [38], [39] and Taiwan [37]. Complete genome sequences that originated in China (available from GenBank) were also included in this analysis, as were the reference sequences for HPV31, HPV33, HPV35, HPV52, HPV58 and HPV67.

### HPV variant lineage classification and nomenclature

The taxonomic grouping and the naming of variant lineages and sublineages were based on the distributions of pairwise comparisons of variant genomes for each HPV type (Figure S1). A similar strategy was previously used for classification of HPV types, species and genera [3]. The distribution of percent differences between variants revealed a bimodal pattern (Figure S1A). This bimodal distribution of individual comparisons indicates that there are variants that are closer related to some variants but not others, thus supporting the grouping of major lineages for each type. Examination of phylogenies for each type (see data presented below) combined with an approximate cut off of 1.0% difference between genomes was used to define major variant lineages. Each major lineage was named using an alphanumeric, with the “A” clade always containing the reference genome for each type. The robustness of this system was examined by viewing the distribution of pairwise comparisons within each variant lineage (i.e., intra-lineage) or between variant lineages for each HPV type (i.e., inter-lineage) (Figure S1B). The overlap between these distributions (0.7%–0.9%) is the reason a fixed value cannot be used to distinguish variant lineages, we conservatively suggest 1.0%, with the caveat that no classification system can exactly classify the process of millions of years of evolution. A similar pattern was seen for the distribution between and within the genome comparisons of sublineages for each HPV type (Figure S1C). Differences between genomes in the 0.5%–1% range were designated as sublineages (e.g., A1, A2, etc.).

### Genomic diversity of HPV31 variants

Three hundred and sixteen HPV31 isolates had the URR and/or E6 regions sequenced (Table 1). These sequences clustered into three main clades from which 22 samples, selected for maximum diversity, were used for complete genome analyses. A total of 299 / 7967 (3.8%) nucleotide positions showed variations compared to the prototype sequence [57] (variable nucleotide sequence positions for each sample are shown in Figure S2A). There were 109 / 2302 (4.7%) encoded variable amino acids (Table 2). The maximum nucleotide pairwise difference between the most dissimilar isolates was 1.4% (Table 2). The most variable region was the noncoding region 2 (NCR2) between E2 and E5 ORFs with 10.2% overall nucleotide diversity (Table 2). One isolate, QV14117, has a 3 bp deletion (ACA) at nt. 1315–1317, resulting in the loss of a threonine at aa 152 within the E1 ORF (Figure S2A).

**Table 2:** Calculation of HPV31, 33 and 35 variations by genome region and open reading frame (ORF).

Genome region and ORF	Maximum nuc. pairwise difference	Number of nucleotide sequences a	Number of variable nuc. positions b		Number of amino acids c	Number of variable aa positions d	
			n	%		n	%
**HPV31** (n = 23)							
E6	2.0%	450	18	4.0%	149	9	6.0%
E7	2.4%	297	15	5.1%	98	10	10.2%
E1	1.1%	1890	48	2.5%	629	22	3.5%
E2	0.9%	1119	31	2.8%	372	17	4.6%
E4	1.6%	309	10	3.2%	102	8	7.8%
NCR1							
E5	3.1%	255	13	5.1%	84	8	9.5%
NCR2	6.6%	118	12	10.2%			
L2	1.5%	1401	50	3.6%	466	24	5.2%
L1	1.4%	1515	55	3.6%	504	19	3.8%
URR	3.2%	953	55	5.8%			
CG e	1.4%	7967	299	3.8%	2302	109	4.7%
**HPV33** (n = 21)							
E6	2.2%	450	13	2.9%	149	8	5.4%
E7	2.0%	294	7	2.4%	97	4	4.1%
E1	0.8%	1935	31	1.6%	644	15	2.3%
E2	1.3%	1062	23	2.2%	353	17	4.8%
E4	2.4%	252	6	2.4%	83	4	4.8%
NCR1	2.3%	43	1	2.3%			
E5	0.9%	228	4	1.8%	75	3	4.0%
NCR2	2.3%	131	3	2.3%			
L2	1.1%	1404	26	1.9%	467	12	2.6%
L1	1.2%	1500	31	2.1%	499	10	2.0%
URR	2.6%	924	48	5.2%			
CG e	1.1%	7912	186	2.4%	2284	69	3.0%
**HPV35** (n = 24)							
E6	1.6%	450	13	2.9%	149	5	3.4%
E7	1.0%	300	3	1.0%	99	2	2.0%
E1	0.3%	1914	14	0.7%	637	9	1.4%
E2	0.9%	1104	18	1.6%	367	15	4.1%
E4	1.4%	291	6	2.1%	96	2	2.1%
NCR1							
E5	0.8%	252	4	1.6%	83	2	2.4%
NCR2	3.5%	145	5	3.4%			
L2	0.9%	1410	23	1.6%	469	9	1.9%
L1	0.7%	1509	24	1.6%	502	4	0.8%
URR	1.6%	879	36	4.1%			
CG e	0.6%	7908	140	1.8%	2306	46	2.0%

a The number of nucleotides within each ORF and region of the genome based on one genome size for each HPV type calculated from the global sequence alignments. Range of values and location in the papillomavirus genome are shown in Figure S4;

b Total number and percentage of positions based on one genome size for each HPV type calculated from the global sequence alignments. Nucleotide variations include SNPs and indels;

c Maximum number of encoded amino acids for each ORF;

d Total number and percentage of variable amino acids based on the maximum number of amino acids derived from the established genome size for each HPV type;

e CG, complete genome. Each nucleotide position is counted once. A single genome size is inferred from the nucleotide alignment (indel was treated as one event).

NCR1, non-coding region 1 (between E2 and E5 ORFs); NCR2, non-coding region 2 (between E5 and L2 ORFs); URR, up stream regulatory region (between stop codon of L1 and start codon of E6).

Phylogenetic trees generated from the complete genome nucleotide sequences clustered HPV31 variants into three distinct lineages designated A, B and C (Figures 1 and 2). As previously described, the HPV31 prototype (HPV31.REF) was assigned to the A lineage (Figure 2). Lineage C was relatively distant to lineages A and B with mean differences of 1.2%±0.11% and 1.2%±0.12%, respectively (Table S2). Phylogenetic trees inferred from the early vs. late regions of the genome showed similar topologies (Figures S3A and S3B). Within the HPV31 genomes, 74 variable nucleotide positions are lineage specific (Figure 8). These variations represent ancestral changes specific to each of the 3 different lineages that have evolved from their common ancestor.

### Genomic diversity of HPV33 variants

Isolates from 179 samples containing HPV33 were classified by sequencing the URR/E6 region and constructing phylogenetic trees (Table 1). Twenty independent complete genomes were sequenced that captured the maximum inter-lineage and intra-lineage heterogeneity based on the URR/E6 region variability. The overall nucleotide and amino acid diversity of the complete genomes were 2.4% (186 sites among 7912 nt) and 3.0% (69 sites among 2284 aa), respectively (Table 2 and Figure S2B). HPV33 isolates QV06895 and QV23819 were the most distantly related genomes with a nucleotide sequence difference of 1.1%; this distance represented the maximum inter-lineage diversity of HPV33 variants (Table S2).

As shown in Figure 3, the topology of the tree constructed with HPV33 variants revealed two distinct lineages, termed A and B. Lineage A was relatively variable and was further divided into two sublineages A1 and A2, that were 0.6%±0.07% dissimilar. These two sublineages were equally distant to the B lineage, with a difference of 0.9%±0.09% nucleotides (Figure S2B, Table S2).

Lineage and sublineage specific nucleotide variations were determined across the complete genome (11 changes for the sublineage A1, 12 for A2, and 38 for B) (Figures 8 and S2B). Insertion and/or deletion (indel) events were detected within the NCR2 and URR regions. Sublineage A1 variants had a long insertion of 79 bp (nt. 7583–7661) within the URR region (Figure S2B).

### Genomic diversity of HPV35 variants

We sequenced the URR/E6 region of 214 HPV35 samples and selected 23 isolates representing each unique variation pattern for complete genome analyses (Table 1). Nevertheless, all variants were highly conserved; the maximum pairwise difference was 0.6% (QV19086 *vs* QV29782) (Table 2 and Figure S2C), and the overall nucleotide diversity was 1.8% (140/7908) (Table 2). In total, 46 / 2306 (2.0%) variable aa positions were detected (Table 2). It should be noted that the isolates analyzed in this study covered the majority of previously reported variations and may represent the maximum genomic heterogeneity of HPV35 variants (Figure 9) [23], [24], [26].

Although HPV35 variants clustered into two clades (Figure 4), the 0.5%±0.06% inter-clade mean difference did not support classification into separate variant lineages (Table S2). Thus, HPV35 variants were divided into two sublineages, A1 and A2. Fourteen sublineage specific nucleotide variations were identified, 7 located within the L2 ORF (Figure 8).

### Genomic diversity of HPV52 variants

Amplification and sequencing the URR/E6 region of 481 samples containing HPV52 resulted in the selection of 22 isolates for complete genome analyses (Table 1). In total, 354 nucleotide sites were variable across the 7993 bp HPV52 genome (4.4%) (Table 1). Of the 2326 encoded amino acids, 105 (4.5%) were variable (Table 3). The maximum nucleotide diversity was 2.2% observed between isolates QV00615 and QV07294. Other features of HPV52 genomes included- the NCR2 and URR regions contained indels, and the L1 ORF had a 3-bp insertion (GGG) between nt. 6191 and 6192 within isolates QV12377 and QV02575 resulting in an insertion of glycine between aa. 209 and 210 (Figure S2D).

**Table 3:** Calculation of HPV52, 58 and 67 variations by genome region and open reading frame (ORF).

Genome region and ORF	Maximum nuc. pairwise difference	Number of nucleotide sequences^ a^	Number of variable nuc. positions^ b^		Number of amino acids^ c^	Number of variable aa positions^ d^	
			n	%		n	%
**HPV52** (n = 23)							
E6	2.2%	444	20	4.5%	147	5	3.4%
E7	3.3%	297	12	4.0%	98	7	7.1%
E1	1.7%	1941	64	3.3%	646	24	3.7%
E2	2.6%	1104	52	4.7%	367	29	7.9%
E4	3.4%	291	14	4.8%	96	8	8.3%
NCR1	8.4%	83	8	9.6%			
E5	3.5%	225	11	4.9%	74	6	8.1%
NCR2	4.0%	129	8	6.2%			
L2	1.9%	1398	47	3.4%	465	19	4.1%
L1	2.2%	1590	61	3.8%	529	15	2.8%
URR	3.9%	909	70	7.7%			
CG^ e^	2.2%	7993	354	4.4%	2326	105	4.5%
**HPV58** (n = 37)							
E6	1.8%	453	16	3.5%	150	8	5.3%
E7	3.4%	300	18	6.0%	99	12	12.1%
E1	1.3%	1938	74	3.8%	645	37	5.7%
E2	1.3%	1080	41	3.8%	359	23	6.4%
E4	2.2%	279	17	6.1%	92	14	15.2%
NCR1	3.2%	62	4	6.5%			
E5	3.5%	234	15	6.4%	77	3	3.9%
NCR2	5.8%	122	14	11.5%			
L2	2.4%	1422	80	5.6%	473	36	7.6%
L1	2.2%	1578	74	4.7%	525	29	5.5%
URR	3.2%	806	72	8.9%			
CG^ e^	1.7%	7837	400	5.1%	2328	148	6.4%
**HPV67** (n = 8)							
E6	1.3%	450	8	1.8%	149	3	2.0%
E7	1.7%	300	7	2.3%	99	4	4.0%
E1	1.3%	1911	30	1.6%	636	9	1.4%
E2	1.1%	1113	19	1.7%	370	10	2.7%
E4	1.7%	300	6	2.0%	99	4	4.0%
NCR1							
E5	1.4%	222	3	1.4%	73	2	2.7%
NCR2	2.6%	117	6	5.1%			
L2	0.9%	1398	18	1.3%	465	11	2.4%
L1	0.8%	1590	24	1.5%	529	6	1.1%
URR	1.8%	801	20	2.5%			
CG^ e^	1.1%	7825	135	1.7%	2321	45	1.9%

a – e see footnotes for Table 2.

Phylogenetic trees inferred from the complete genome nucleotide sequences separated HPV52 variants into four distinct lineages A, B, C and D (Figure 5). The lineages A, B and C form one clade, and are 0.8%–1.1% different among themselves, and distantly separated from lineage D (mean differences of 1.8%–2.0%) (Table S2). The deep separation between lineages C and D (2.0%±0.14% different) is similar to that observed between HPV16 variant lineages E (As) and AA (1.9%±0.15% different) [11] and HPV18 variant lineages Af and non-Af (2.0%±0.16% different) [12].

HPV52 variants within lineages B and C were further divided into sublineages, B1 and B2 (0.6%±0.08% different), and C1 and C2 (0.6%±0.07% different) (Figure 5 and Table S2). There were 97 nucleotide changes specific for lineage D, while lineages A, B and C have 15, 8, and 18 unique distinguishing nucleotide variations, respectively (Figures 8 and S2D). There were multiple indels in the NCR2 and URR regions and an insertion of GGG encoding glycine within the L1 ORF of isolates Qv12377 and Qv2575 (Figure S2D). The noncoding regions (NCR1, NCR2 and URR) were most variable in overall nucleotide diversity, followed by E5/E4/E7, E6/L1, and L2/E1 (Table 3).

### Genomic diversity of HPV58 variants

The URR/E6 partial sequences clustered 447 HPV58 variants into four major clades; 26 isolates capturing the maximum viral genomic heterogeneity were amplified and sequenced (Table 1). In addition, 10 HPV58 variant complete genomes from China were available from GenBank (listed in Table S1) and were included in the complete genome analyses. In total, 400 variable nucleotide positions were identified within the 7837 bp HPV58 genome (5.1%) (Table 3). There were 148/2328 variable amino acids (6.4%) (Table 3, Figure S2E). There was a 1.7% maximum pairwise nucleotide difference between isolates QV03554 and RW063. Three isolates, QV03841, QV03858, QV04732 had a 3-bp deletion within the E2/E4 ORF (nt. 3527 – 3529 of E2 and nt. 3525 – 3527 of E4), resulting in the loss of a glycine (aa. 259 of E2) and a glutamic acid (aa. 67 of E4) (Figure S2E).

Phylogenetic topology and percent nucleotide differences between clades classified HPV58 variants into four lineages (A, B, C and D) that are further parsed into seven sublineages (A1/A2/A3, B1/B2 and D1/D2) (Figure 6). The inter-lineage mean difference of HPV58 variants ranged from 0.9%–1.4%, and the inter-sublineage differences were 0.5%–0.7% (e.g., A1 *vs* A2) (Table S2). Although sublineages B1 and B2 did not cluster together in the complete genome tree (Figure 6), both were more closely related to each other (0.7%±0.07% different) than to other lineages/sublineages (>1.0% mean differences) (Table S2). One hundred and forty nucleotide changes are lineage- and/or sublineage- specific (Figures 8 and S2E). All 447 HPV58 isolates from our laboratory had a C307T change within E6; whereas, except for TJ18_58, all HPV58 isolates from Chinese patients had a cytosine (C) at nt. 307 identical to the prototype [58] (Figure S2E). These later variants form sublineage A1 (Figure 6).

### Genomic diversity of HPV67 variants

HPV67 has a low prevalence throughout the world making accurate assessment of its variability and oncogenicity difficult. There were a total of 32 cervicovaginal samples containing amplifiable HPV67, of which 7 complete genomes representing different variant patterns were characterized (Table 1). There were 135 nucleotide changes across the 7825 bp genome (1.7%), and 45 / 2321 amino acid positions were variable (1.9%) (Table 3). The nucleotide sequence difference between isolates QV22701 and QV24827 (1.1% difference) represents the maximum inter-lineage diversity (Table 3 and Figure S2F).

The eight HPV67 variant genomes formed two distinct lineages, termed A and B, that were 1.0% different from each other (Figure 7 and Table S2). Lineage A was further subdivided into sublineages A1 and A2. The HPV67 prototype differed from all other variants by >0.3% based on pairwise comparisons. There were 16 nucleotide changes conserved among the 7 newly sequenced HPV67 isolates that were different than the prototype genome; it is unknown whether these polymorphisms are natural variations or errors in the original prototype sequence. Indel events were detected within the E2/E4, NCR2 and URR regions (Figure S2F).

### HPV16-related alpha-9 HPV genomic diversity

To estimate the coverage of type specific SNP variation within the sample cohorts and genomes analyzed, rarefaction curves of the single-nucleotide polymorphisms (SNPs) were plotted (Figure 9). Based on the analysis of conserved SNP sites (i.e., SNPs detected in ≥2 samples), the plot for the combined genomes for each type suggests that sampling of genomes within the targeted populations may increase the repertoire of genomic variability. However, it is unlikely to reveal novel variant lineages, since the curves flatten out with increasing numbers of sequenced genomes. The pairwise inter-lineage mean differences revealed maximum genomic diversity of HPV52 and HPV58 isolates, followed by HPV31, HPV33 and HPV67 variant genomes (Table S2). HPV35 isolate genomes were not highly variable and only a single lineage was observed.

When each ORF/region was compared, the noncoding regions (NCR1, NCR2 and URR) most often showed the largest variability, followed by the E5 and E4/E2 overlap ORFs (Tables 2 and 3). The diversity of the E6, E7, E1, E2 (taken in its entirety), L2 and L1 ORFs varied by type; nevertheless, the L1 ORF was not significantly more conserved in terms of the overall nucleotide diversity compared to the other ORFs (Tables 2 and 3).

### Comparison of early and late gene phylogenies

The topology of phylogenetic trees constructed from different genes or regions of genomes can indicate differences in evolutionary history. We previously demonstrated that phylogenetic incongruence exists between trees inferred for late and early regions of the *Alphapapillomaviruses* [11], [51]. To evaluate whether similar evolutionary patterns could be identified in the HPV16-related viral genomes, we compared trees created with the early region ORFs (i.e., E1, E2, E5, E6 and E7) to that of the late ORFs (i.e., L1 and L2) (Figure S3, panels A and B). At the type level, HPV16, HPV31 and HPV35 isolates formed a consistent clade with HPV31 and HPV35 sharing a most recent common ancestor (MRCA), as did the HPV33 and HPV58 isolates. The position of HPV52 and HPV67 isolates varied slightly; only in the tree using the L1/L2 did HPV52 and HPV67 isolates show evidence of having shared a MRCA (Figure S3, panels A and B). Comparison of trees for the early and late regions indicated some minor changes of the topology of variant lineages, as shown in Figure S3.

## Discussion

In this work, over 12,000 cervicovaginal samples from women in the Americas (Costa Rica), Africa (Rwanda, Zambia, and Burkina Faso) and Asia (China, Taiwan, and Indonesia) were tested for HPV and 120 genomes from nearly 2,000 HPV16-related alpha-9 HPV isolates (HPV31, 33, 35, 52, 58 and 67) had their complete genomes sequenced. These HPV isolates were selected based on the analysis of the URR/E6 regions to identify samples representing or forming major variant lineages and also having the most diverse URR/E6 regions for each type [23], [24], [25], [26], [27], [28]. Based on the analyses of these genomes, there are two aspects of this study that deserve further consideration. First, the descriptive aspect of the HPV16-related alpha-9 type variants provide a framework to establish a nomenclature for variant lineages. Second, an emerging picture of the evolution of this highly pathogenic clade (see Figure 1) of HPVs is discussed.

Isolates of the same HPV type were originally considered as “variants” when their L1 genes contained 1 to 2% nucleotide sequence differences [4]; however, the L1 ORF does not contain the optimal sequence information for distinguishing closely related HPV variants. As part of the ICTV Papillomavirus Study Group, we were recently assigned the task of developing a classification system for HPV variants [3]. In contrast to the genera, species and type definitions that are based on the L1 ORF nucleotide sequence, we set the criteria for classification and nomenclature of variant lineages and sublineages using the complete genome, since the recently evolved variant genomes have changes that are not always evenly distributed throughout the genome (see Figure 8). To define distinct variant lineages, we used a nucleotide sequence difference of approximately 1.0% between two or more variants of the same type. This value was derived from empiric data on the distribution of differences between genomes of the same type (see Figure S1). Similarly, differences across the genome of 0.5%–1.0% were used to identify sublineages. Each variant lineage was classified and named with an alphanumeric value (see Figure 1 for summary). The prototype sequence (i.e., the cloned genome designated as the original type) is always designated variant lineage A and/or sublineage A1 [12].

Variants of HPV31, 33, 52, 58 and 67, similar to HPV16 and HPV18, form at least two deeply separated clades suggesting codivergence of host and virus as different lineages diversified from their most recent common ancestor (MRCA) [11], [12]. HPV35 variants are highly conserved and did not meet criteria for classification into more than one lineage. This probably represents a recent divergence from the MRCA of the HPV31, HPV35 and HPV16 clade. Alternatively, another variant lineage of HPV35 might exist in an isolated and/or unsampled population or could have disappeared by genetic isolation and/or host demise.

Although HPV16 and HPV18 variants are associated with specific geographic locations, the geographic distribution and ethnic association of HPV31, 33, 35, 52, 58 and 67 variant lineages are not well established. We believe a nomenclature based on alphanumerics is preferable to one based on geographic names, since it eliminates the problem of naming a lineage found in multiple geographic areas.

A number of investigators have used the strategy of PCR amplifying and sequencing one or a few informative segments to classify isolates into different variant lineages or groups. For example, HPV58 variants containing E7 SNPs- C632T and G760A (aa 63G) that have been reported to be associated with higher cervical cancer risk [34] can be classified into HPV58 sublineage A3 (Figure S2E). The C7732G SNP in HPV33 variants, which results in the loss of a putative binding site for the cellular upstream stimulatory factor has also been associated with high-grade squamous intraepithelial lesions (HSILs) [33]. HPV33 C7732G is a lineage specific SNP within the URR region and represents HPV33 variant lineage A2 (Figure S2B). The URR region contains many *cis*-acting regulatory sequences; variations within these motifs may alter viral transcription and replication. Alternatively, these changes may be markers of other linked nucleotide changes within a lineage. A few studies have reported that alpha-9 HPV variants differ in risk of persistence; for some HPV genotypes, variant lineages or sublineages (e.g., HPV35 A1) differ in their risk of CIN3+ [26], [43]. Knowledge of the complete genome sequences and phylogenetic structure will facilitate understanding the clinical role sequence variations play in genotype-phenotype associations. An important point of the current analysis is showing that individual or groups of SNPs need to be interpreted in light of the high correlation of sets of SNPs within each lineage (Figure 8). We have previously termed the stochastic process of papillomavirus genome accumulated mutations, “lineage fixation” [11]. This has important practical considerations in that investigators sequencing or analyzing different regions of the genome will now be able to classify the lineages of these variants for genotype-phenotype studies based on the sequence data presented in Figure S2. Thus, a common nomenclature will allow HPV researchers to discuss the properties of HPV variant lineages without having to describe sets of nucleotide changes to define a group of HPV variants. This will be particularly useful for future studies of the alpha-9 species group of HPVs that is an abundant and related group of viruses that have a high pathogenic potential.

Papillomavirus genomes accumulate SNPs and indels (see Figure S4) through a stochastic process based on mutation rates similar to the host genomes they infect [59]. This reflects the fact that PVs use the host's DNA replication machinery to copy and amplify their genomes; natural selection has likely played an important role over the course of evolution to filter and fix nucleotide changes within variant lineages. Whether the variation seen in highly related genomes (i.e., the variants described in this report) are determined by selection or genetic drift remain to be determined. However, the relatively recent evolution of the alpha-9 group of HPV types and variants cannot easily be explained by natural selection. Analyses used to detect selection at individual codon position [60] identified only a few scattered nucleotide sites under Darwinian selection. This might be expected in viruses that have existed for hundreds of millions of years [61], and have perfected a survival strategy and optimized their structural components via natural selection [61], [62]. This inference is supported by the existence of strong purifying selection and conserved genome regions that are most evident in the L1, L2, E1 and E2 ORFs. Other regions of the genome have more flexibility to adapt HPVs to different biological niches, but the exact mechanisms and sequences responsible for these changes have not been identified. Moreover, since recombination is not a major form of papillomavirus evolution, SNPs are not correlated by distance, as is observed in the human hapmap and 1000 genome projects resulting in linkage disequilibrium (LD) blocks [63], [64]. In contrast, HPV evolution results in genome variation where changes in one region of the genome are highly correlated with those in other regions of variants from the same lineage (Figure 8). Nevertheless, there is at least one example of recombination between a polyomavirus and a papillomavirus [65] indicating that recombination has occurred in the distant past and could occur in the future. To date, our laboratory has not observed direct evidence of recombination in human papillomavirus genomes, and there is a lack of compelling data to suggest that recombination is important in the evolution of the alpha-9 HPVs.

In summary, we present an extensive description of the HPV16-related alpha-9 papillomavirus variants. We provide a taxonomy and nomenclature of these variants that should be useful for evolutionary biologists, virologists, epidemiologists and health care workers. Nevertheless, the mechanisms of adaptation and oncogenic pathogenicity of the alpha-9 HPVs will require additional studies and their role in morbidity and mortality, especially for cervix cancer, will continue for decades to come.

## Supporting Information

Figure S1 **Distribution of pairwise differences between nucleotide sequences of HPV16-related alpha-9 type genomes.** The genome nucleotide sequences of each type were globally aligned using the program MAFFT v6.846 [46]. The p-distance method in the MEGA5 [54] was used to calculate the percent differences for each isolate comparing to all other isolates of the same type based on a global alignment. The Y-axis represents the number of comparisons. The X-axis shows the percent nucleotide pairwise differences. (A) Comparison of each isolate to all other isolates of the same type, resulting in a total of 1686 values. (B) Inter- and intra-lineage pairwise differences. Inter-lineage: comparisons of isolates within different lineages of the same type (894 comparisons). Intra-lineage: comparisons of isolates within the same lineage (790 comparisons). (C) Inter- and intra-sublineage pairwise differences. Inter-sublineage: comparisons of isolates within different sublineages of the same lineage (338 comparisons). Intra-sublineage: comparisons of isolates within the same sublineage (452 comparisons).
(PDF)

Figure S2 **Variation at nucleotide and amino acid positions within the complete genomes and ORFs of HPV16-related alpha-9 isolates.** Amino acids alignments were used to guide the nucleotide sequence alignments as previously described [12]. The original Genbank sequence for each type is used as the reference for all alignments and is shown at the top of each panel. Only sites that are different are displayed. Below the nucleotide sequence alignments are the corresponding amino acid differences for each ORF. The nucleotide sequence variations are shown for each position listed at the top of the panel by ORF or region. Under the reference sequence the nucleotide sequence of each isolate is displayed listing only sites that are different from the reference sequence in one or more of the isolates (name is on the left of the panel with the type|sample identifier|lineage or sublineage listed). Dots, sites matched with reference sequence; dashes, indel events. NCR1, noncoding region between E2 and E5 ORFs; NCR2, noncoding region between E5 and L2 ORFs; URR, upstream regulatory region located between stop codon of L1 and start codon of E6. Genome sequences for each lineage or sublineage are alternatively shown as grey blocks for visualization of most closely related isolates. (A) alignment of HPV31 complete genomes, (B) alignment of HPV33 complete genomes, (C) alignment of HPV35 complete genomes, (D) alignment of HPV52 complete genomes, (E) alignment of HPV58 complete genomes, and (F) alignment of HPV67 complete genomes.
(PDF)

Figure S3 **Early and late gene tree comparison.** The trees were constructed using the MrBayes (v3.1.2) program based on the concatenated nucleotide sequences of “early genes” (E6, E7, E1, E2 and E5) (Panel A) and “late genes” (L2 and L1) (Panel B). To root the tree, HPV34 and HPV73 prototype sequences (NCBI accession numbers X74476 and X94165) were set as the outgroup and are represented by grey broken lines. The shaded areas represent groupings of lineages and sublineages of HPV16, HPV31, HPV33, HPV35, HPV52, HPV58 and HPV67. The length of broken and solid lines represent distance between clades, although the number of changes is different for these two lines, the scale is indicated in the upper left corner of the figure.
(PDF)

Figure S4 **Representation of an alpha-9 HPV genome and ORF/region length ranges.** Each region or ORF of the HPV genome is indicated outside the double-stranded circle. Lengths of each ORF and region are indicated by the histogram pointing to the region/ORF in the figure. The length in nucleotide sequences (bp) for each HPV16-related alpha-9 HPV genome is indicated with the minimal and maximal lengths represented by the bars with dots or highlighted in grey, respectively. The diagram of the HPV genome is not drawn to scale and the histogram for each ORF/region is presented in a different range of values.
(PDF)

Table S1 **List of HPV genomes by type, isolate name, geographic origin of sample, lineage designation, length of complete genome and NCBI #.**
(PDF)

Table S2 **Nucleotide sequence mean difference (± standard error) of HPV16-related alpha-9 HPV complete genomes.** The intra-lineage (e.g., A vs. A) and intra-sublineage (e.g., A1 vs. A1) difference values are highlighted in gray.
(PDF)
