# Supplementary figures and images for: Evolution and Taxonomic Classification of Human Papillomavirus 16 (HPV16)-Related Variant Genomes: HPV31, HPV33, HPV35, HPV52, HPV58 and HPV67
Source: PLoS One. 2011 May 27;6(5):e20183. doi: 10.1371/journal.pone.0020183 (PMC3103539; doi:10.1371/journal.pone.0020183)

A

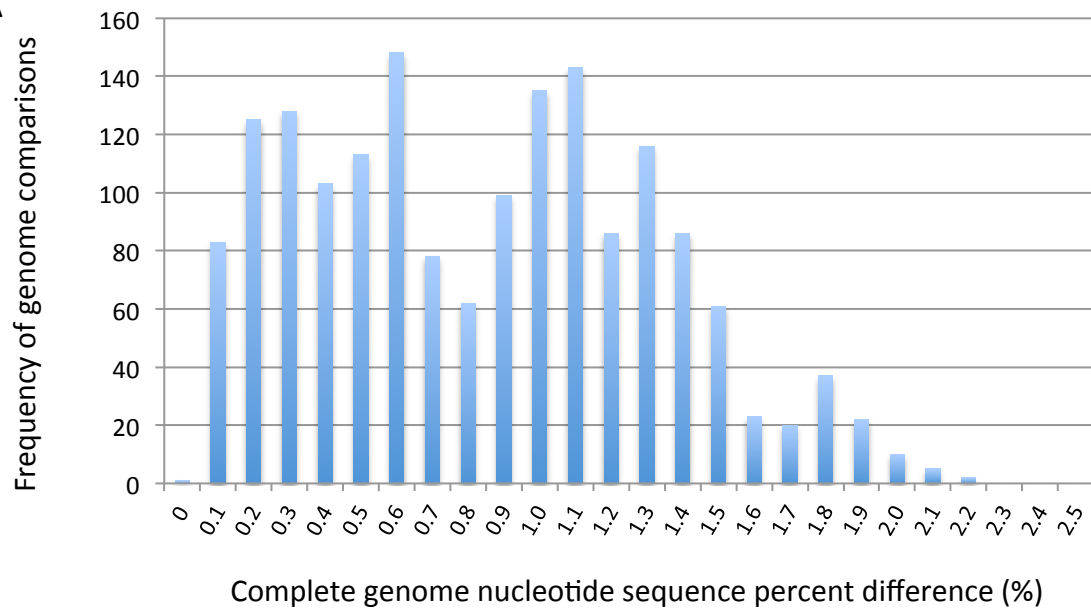

B

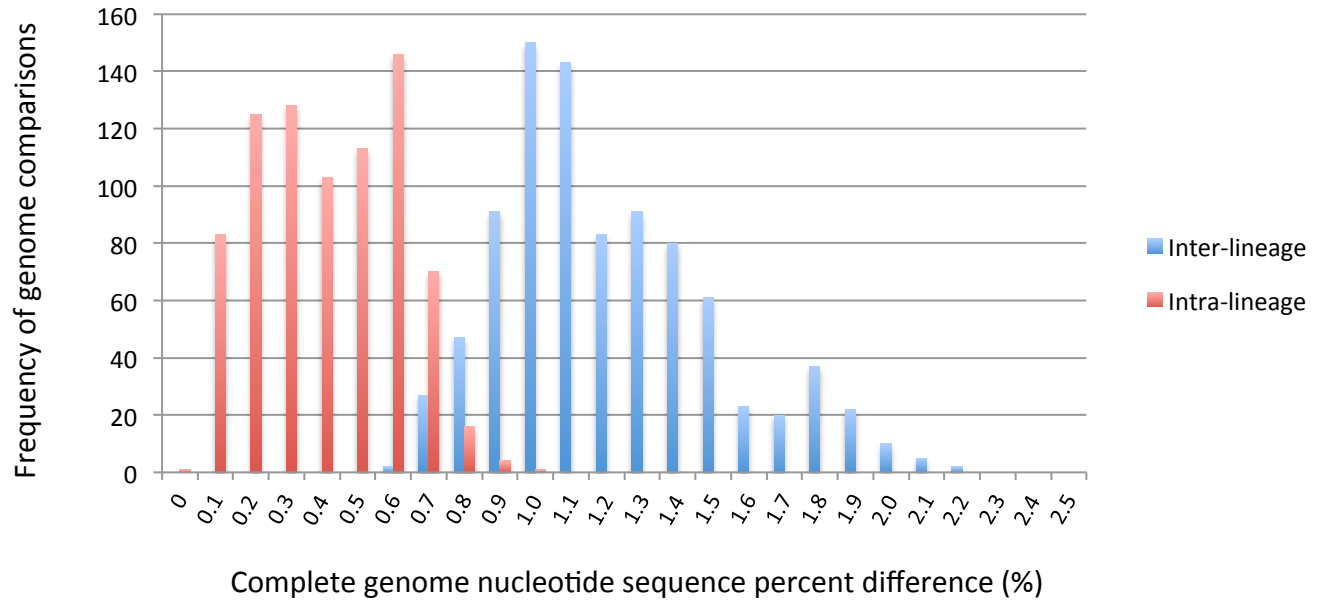

C

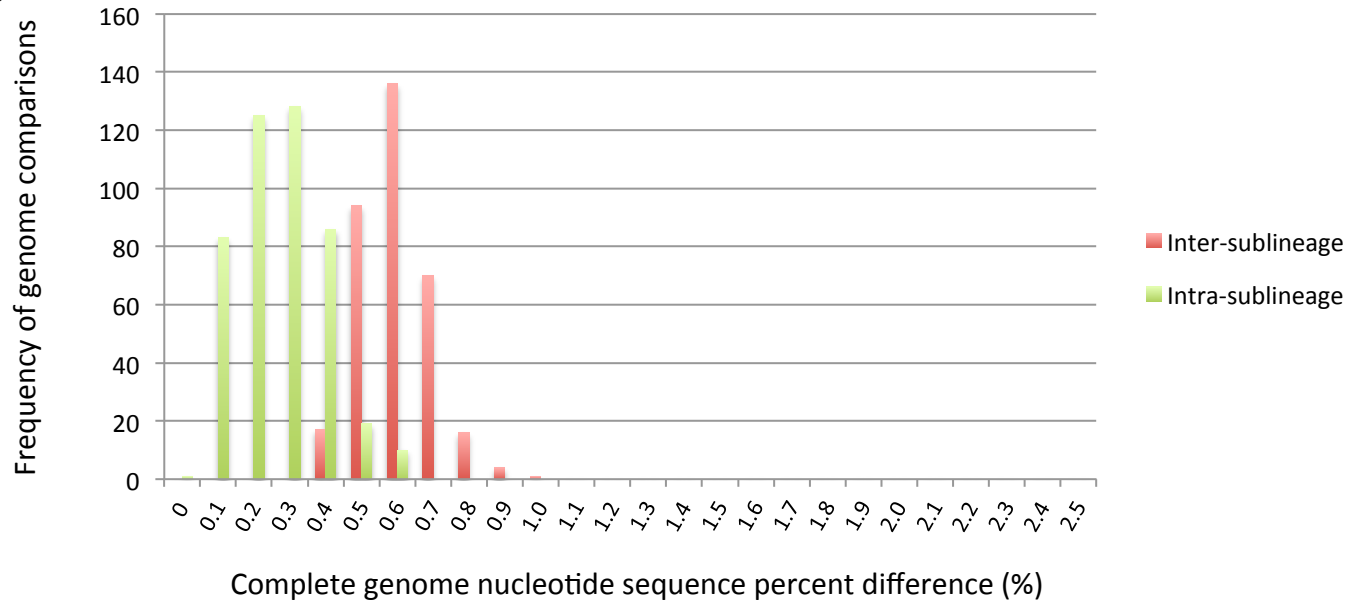

Supplement: Figure S1 — Distribution of pairwise differences between nucleotide sequences of HPV16-related alpha-9 type genomes. The genome nucleotide sequences of each type were globally aligned using the program MAFFT v6.846 [46]. The p-distance method in the MEGA5 [54] was used to calculate the percent differences for each isolate comparing to all other isolates of the same type based on a global alignment. The Y-axis represents the number of comparisons. The X-axis shows the percent nucleotide pairwise differences. (A) Comparison of each isolate to all other isolates of the same type, resulting in a total of 1686 values. (B) Inter- and intra-lineage pairwise differences. Inter-lineage: comparisons of isolates within different lineages of the same type (894 comparisons). Intra-lineage: comparisons of isolates within the same lineage (790 comparisons). (C) Inter- and intra-sublineage pairwise differences. Inter-sublineage: comparisons of isolates within different sublineages of the same lineage (338 comparisons). Intra-sublineage: comparisons of isolates within the same sublineage (452 comparisons). (PDF) [file pone.0020183.s001.pdf]

----- 0.04 changes per site  
—— 0.002 changes per site

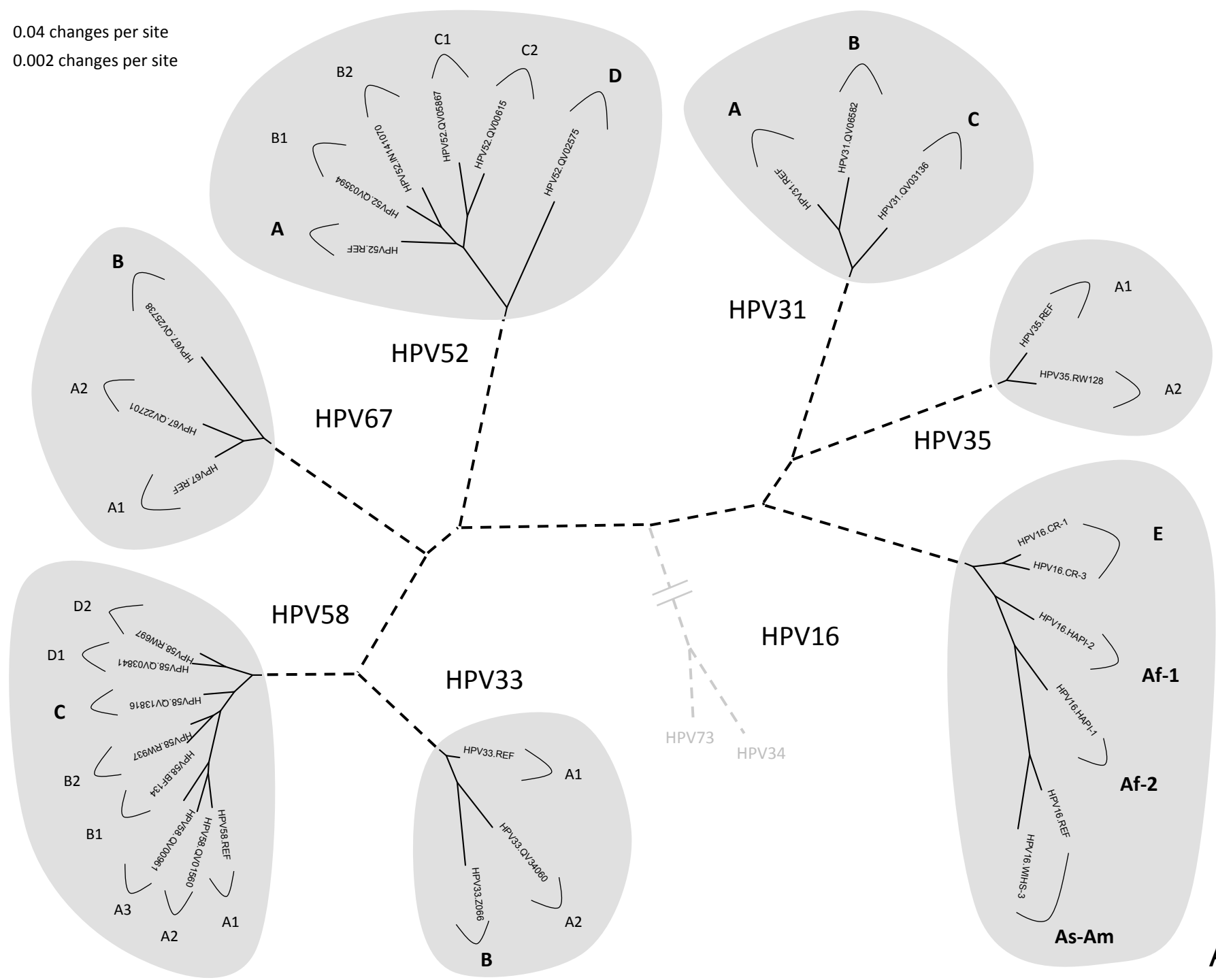

----- 0.04 changes per site  
—— 0.002 changes per site

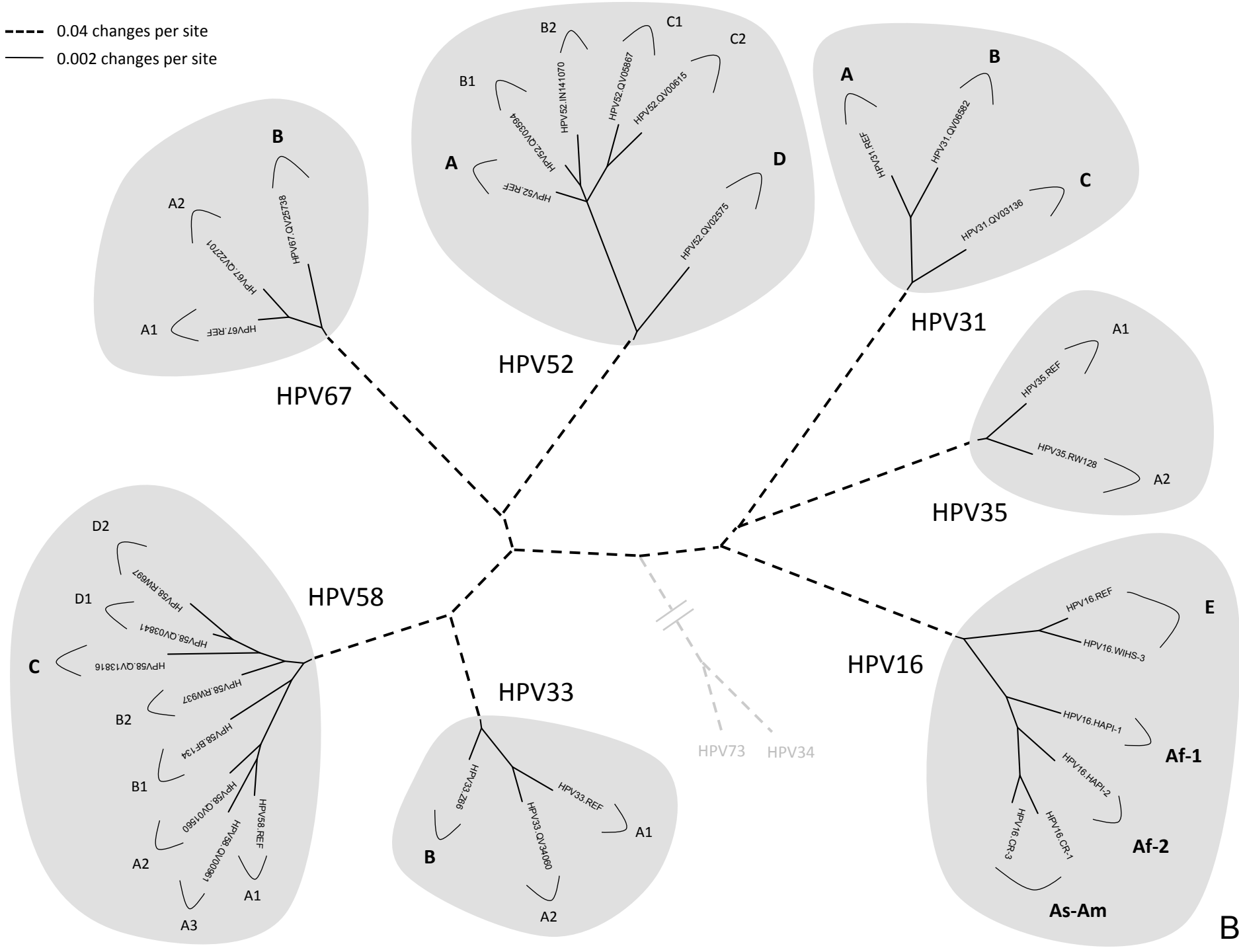

Supplement: Figure S3 — Early and late gene tree comparison. The trees were constructed using the MrBayes (v3.1.2) program based on the concatenated nucleotide sequences of “early genes” (E6, E7, E1, E2 and E5) (Panel A) and “late genes” (L2 and L1) (Panel B). To root the tree, HPV34 and HPV73 prototype sequences (NCBI accession numbers X74476 and X94165) were set as the outgroup and are represented by grey broken lines. The shaded areas represent groupings of lineages and sublineages of HPV16, HPV31, HPV33, HPV35, HPV52, HPV58 and HPV67. The length of broken and solid lines represent distance between clades, although the number of changes is different for these two lines, the scale is indicated in the upper left corner of the figure. (PDF) [file pone.0020183.s003.pdf]

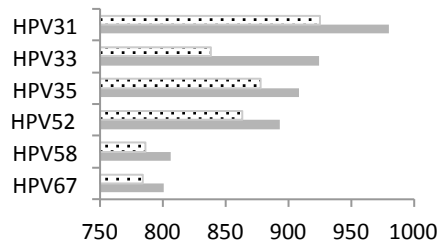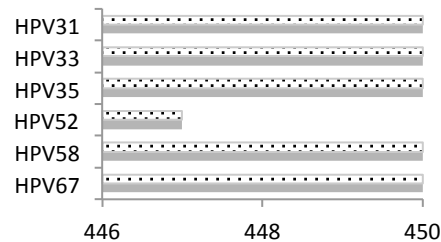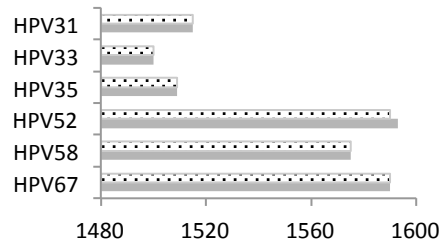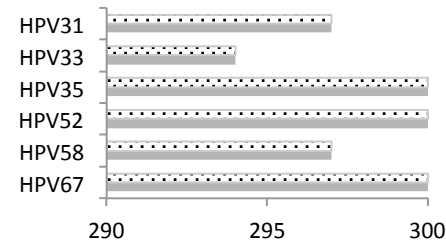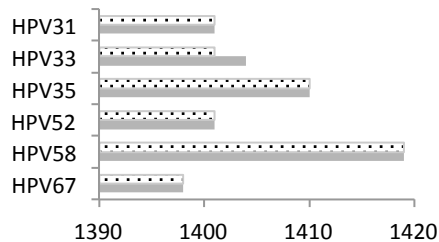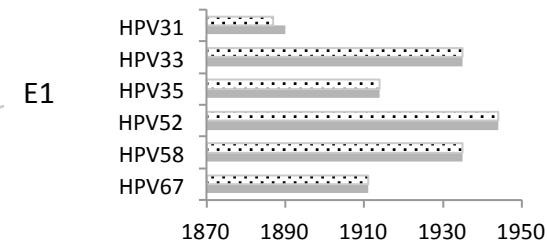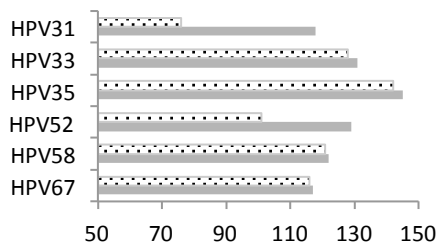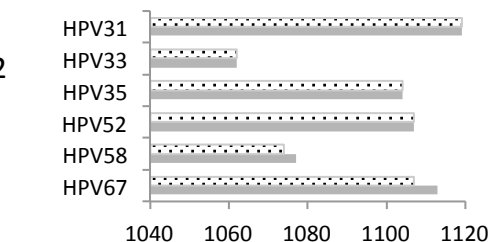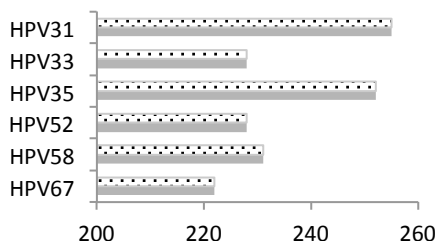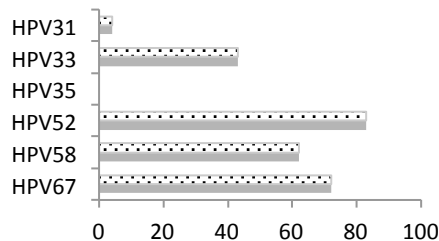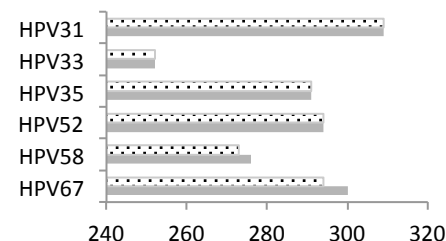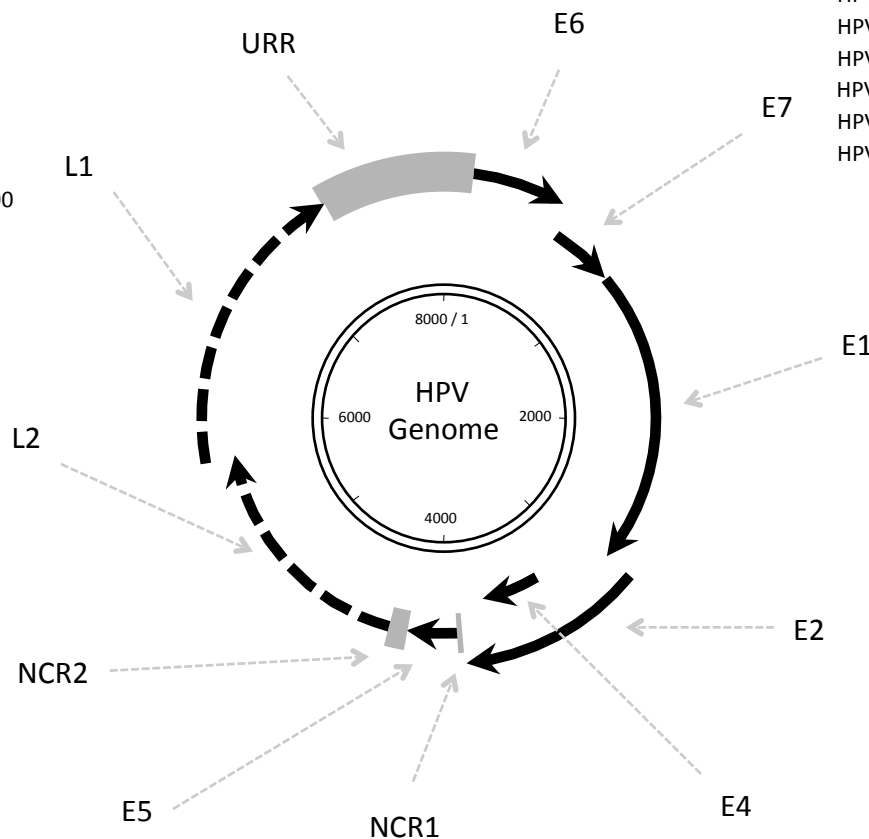

Supplement: Figure S4 — Representation of an alpha-9 HPV genome and ORF/region length ranges. Each region or ORF of the HPV genome is indicated outside the double-stranded circle. Lengths of each ORF and region are indicated by the histogram pointing to the region/ORF in the figure. The length in nucleotide sequences (bp) for each HPV16-related alpha-9 HPV genome is indicated with the minimal and maximal lengths represented by the bars with dots or highlighted in grey, respectively. The diagram of the HPV genome is not drawn to scale and the histogram for each ORF/region is presented in a different range of values. (PDF) [file pone.0020183.s004.pdf]
