# Supplementary material for: Evolution and Taxonomic Classification of Human Papillomavirus 16 (HPV16)-Related Variant Genomes: HPV31, HPV33, HPV35, HPV52, HPV58 and HPV67
Source: PLoS One. 2011 May 27;6(5):e20183. doi: 10.1371/journal.pone.0020183 (PMC3103539; doi:10.1371/journal.pone.0020183)
Supplement: Figure S2 — Variation at nucleotide and amino acid positions within the complete genomes and ORFs of HPV16-related alpha-9 isolates. Amino acids alignments were used to guide the nucleotide sequence alignments as previously described [12]. The original Genbank sequence for each type is used as the reference for all alignments and is shown at the top of each panel. Only sites that are different are displayed. Below the nucleotide sequence alignments are the corresponding amino acid differences for each ORF. The nucleotide sequence variations are shown for each position listed at the top of the panel by ORF or region. Under the reference sequence the nucleotide sequence of each isolate is displayed listing only sites that are different from the reference sequence in one or more of the isolates (name is on the left of the panel with the type|sample identifier|lineage or sublineage listed). Dots, sites matched with reference sequence; dashes, indel events. NCR1, noncoding region between E2 and E5 ORFs; NCR2, noncoding region between E5 and L2 ORFs; URR, upstream regulatory region located between stop codon of L1 and start codon of E6. Genome sequences for each lineage or sublineage are alternatively shown as grey blocks for visualization of most closely related isolates. (A) alignment of HPV31 complete genomes, (B) alignment of HPV33 complete genomes, (C) alignment of HPV35 complete genomes, (D) alignment of HPV52 complete genomes, (E) alignment of HPV58 complete genomes, and (F) alignment of HPV67 complete genomes. (PDF) [file pone.0020183.s002.pdf]

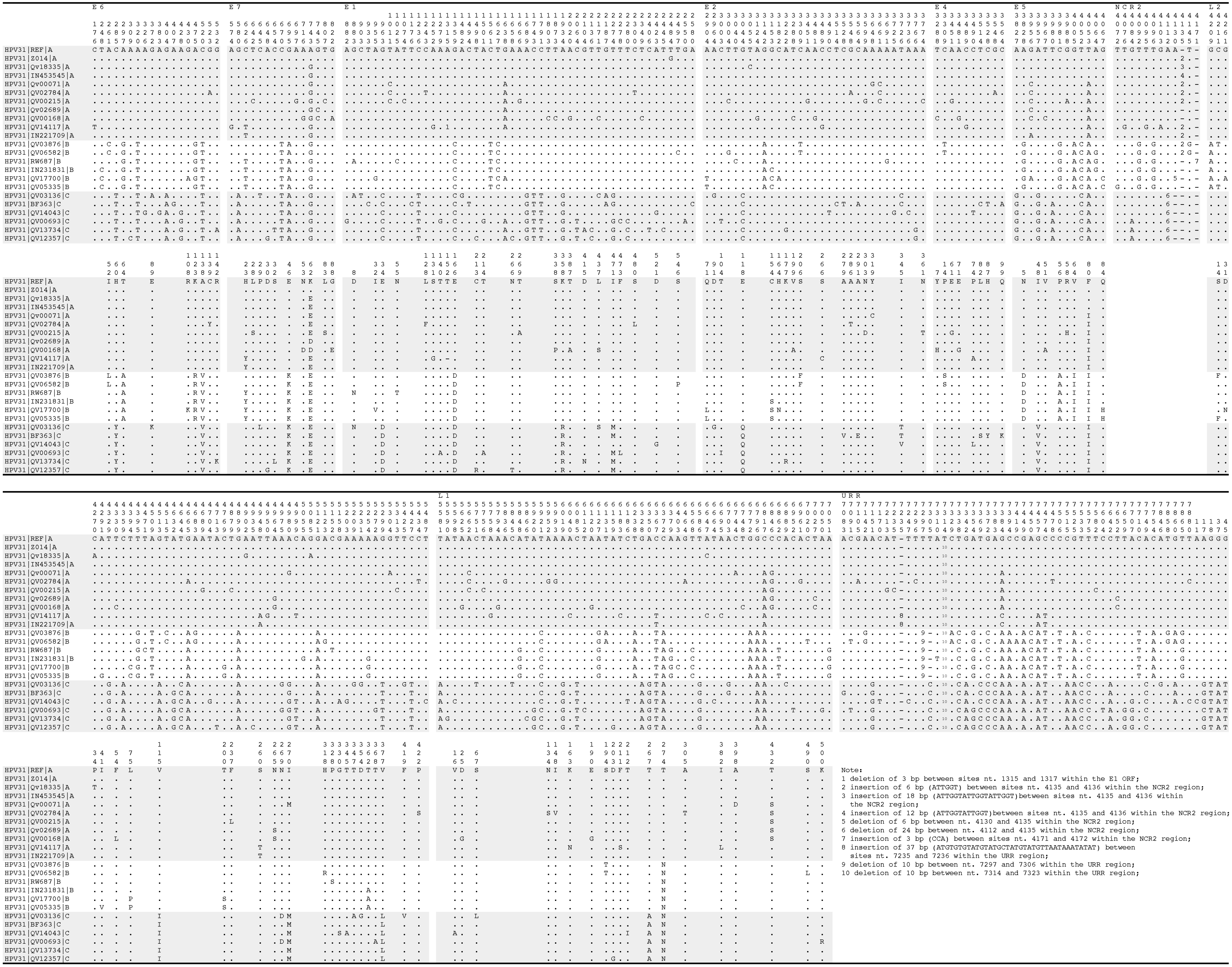
E6 E7 E1 E2 E4 E5 NCR2 L2 L1 URR
HPV31|REF|A
HPV31|Z014|A
HPV31|Qv18335|A
HPV31|IN453545|A
HPV31|Qv00071|A
HPV31|QV02784|A
HPV31|QV00215|A
HPV31|Qv02689|A
HPV31|QV00168|A
HPV31|QV14117|A
HPV31|IN221709|A
HPV31|QV03876|B
HPV31|QV06582|B
HPV31|RW687|B
HPV31|IN231831|B
HPV31|QV17700|B
HPV31|QV05335|B
HPV31|QV03136|C
HPV31|BF363|C
HPV31|QV14043|C
HPV31|QV00693|C
HPV31|QV13734|C
HPV31|QV12357|C
Note:
1 deletion of 3 bp between sites nt. 1315 and 1317 within the E1 ORF;
2 insertion of 6 bp (ATTGGT) between sites nt. 4135 and 4136 within the NCR2 region;
3 insertion of 18 bp (ATTGGTATTGGTATTGGT)between sites nt. 4135 and 4136 within the NCR2 region;
4 insertion of 12 bp (ATTGGTATTGGT)between sites nt. 4135 and 4136 within the NCR2 region;
5 deletion of 6 bp between nt. 4130 and 4135 within the NCR2 region;
6 deletion of 24 bp between nt. 4112 and 4135 within the NCR2 region;
7 insertion of 3 bp (CCA) between sites nt. 4171 and 4172 within the NCR2 region;
8 insertion of 37 bp (ATGTGTGTATGTATGCTATGTATGTTAATAAATATAT) between sites nt. 7235 and 7236 within the URR region;
9 deletion of 10 bp between nt. 7297 and 7306 within the URR region;
10 deletion of 10 bp between nt. 7314 and 7323 within the URR region;

A

	E6	E7	E1	E2	E4	E5	NCR	L2	L1	URR
			11111111111111111 2222222222	22222333333333333333333	333333	33344	4 4	4444444444444455555555555555	5555555666666666666666666677777	77777777777777777777777777777777777777777
	2223333344555	6777888	8999001122333455788992345556677	78899001222334444466777	344444	89900	1 1	2222345566688911122222222345	7778899012333344556666777900000	11111111111122222334444444444555556777888
	0111256848234	0023356	8479681368579208989576615778646	65769169227190356735225	903567	12716	6 6	2479031207924013612357778253	5675999175399978992367034106777	01234667778922589670122234458333884036778 12899
	7345554790149	6627302	8749512634397779607223991579582	23736309695881689543459	816895	88161	7 7	6773586701045580640070127473	2032007050701687255443478383278	568351924528570552646245623413573411283901689146
HPV33|Ref|A1	CACCAGAAAACCT	GCTAGCA	CCTGACTAGATTGGATAATTTGTAATTGCCC	CGGTGATAACCTAACCACTCGTC	AACCAC	TCTTC	---	CCCATGTAGTCAACAAACCGGCGTGACT	ACACAGAATTCCAGTAGAAAAACTAAGGAAA	ATTCTTGTTTGTAGCTTTCTTGACAGCGT-GCTGCTCATATGCGGAAG
HPV33|Qv32494|A1	....	....	..............G................	......................T	......	..C..	T--	......C...........T.........	................A..............	C...................G........-...............C..
HPV33|Qv22751|A1	....	....	............A...........C......	......................T	......	.....	TTT	..................TA......T.	.........A.............C.......	.............................-A...G..........C..
HPV33|Qv35834|A1	........T....	....	....	......................T	......	...G.	TTT	..................T.........	...........................A...	....C........................-...............C..
HPV33|BF266|A1	....	....	....	......................T	......	.....	---	..................T.........	...........................A...	.............................-...............C..
HPV33|RW702|A1	....	....	....	......................T	......	.....	---	.....A............T.........	...........................A...	..................G..........-...............C..
HPV33|Z084|A1	....	....	........A......................	......................T	......	.....	---	.....A............T.........	....	.............................-...A...........C..
HPV33|RW926|A1	....	....	....	................C.....T	....C.	.....	---	.....A............T.........	....	.............................-...............C..
HPV33|Qv18347|A1	.C...........	...G..T	.......G........G.GC...........	.................T....T	.....T	.....	TT-	.....A.......T....T.A.......	......G.C..A.A.....GGG...G.....	......TG.....A.C.....TG.G...G-.......C.......C.A
HPV33|Qv06895|A1	.C...........	...G..T	....G..G.G......G.GC.......C...	.................T....T	.....T	.....	TTT	..T..A.......T....T.A.......	......G.C..A.A....GGGGT..G.....	......TG.....A.C.....TG.G...G-...........C...C.A
HPV33|AS099|A1	.C.....C....C	.A.....	.......G.......CG..C...........	.........T.......T....T	.....T	.....	---	.T...A.......T....T.........	......G....A.A......G..........	......T..-...AT......TG......-...............C..
HPV33|Qv34060|A2	.C....C..T...	...G..T	......CG.....A..G..C...T....T..	..T.....G.T..C..CT.GTCT	.C..CT	....T	T--	.....A...C...T.G..T.C...AC..	.A...A.....A..C.....G...C......	...T.........A.....A.T....TA.-AA222.G..G..GA.C..
HPV33|INCC0137|A2	.C....C..T...	...G..T	......CG.....A..G..C........T..	..T.....G.T..C..CT.GTCT	.C..CT	....T	---	.....A...C...T.G.TT.C...AC..	GA...A.....A..C.....G..........	.............A.....A.T....TA.-AA222.G..G..GA.C..
HPV33|INJP06456|A2	.C....C..T...	...G..T	......CG.....A..G..C........T..	..T.....G.T..C..CT.GTCT	.C..CT	....T	---	.....A...C...T.G..T.C...AC..	.A...A.....A..C.....G..........	.............A.....A.T....TA.-AA222.G..G..GA.C..
HPV33|Qv32431|A2	.C....C..T...	......T	T..T..CG........GG.C......C.T.T	T.TC....G....C..CT.GTCT	.C..CT	.....	TTT	.....A...C...T.G..T.C...AC..	.A.T.A.....A..C.....G.....A....	.............A.....A.T.....A.-AA222AG.....GA.C..
HPV33|RW136|B	...TCT.C..TT.	A...TGT	..........A.....G..CC.G..C...G.	..T..G.GG.....TG...GTCT	..TG..	.T...	---	TT.TCA..A..TTT..G.T.C....C.C	.AG.GA....TA...T......T.....GGG	.GC.....C.CC.A...C.A.T.T.A.AG1A.222........A.CG.
HPV33|IN241890|B	...TCT.C..TT.	....TGT	.....T..........G..CC.G..C...G.	..T..G.GG.....TG..CGTCT	..TG..	.T...	---	...TCA..A..TTT..G.T.C....C.C	.AG.GA....TA...T......T......GG	.GC.....C.CC.A...C.A.T.T.A.AG-A.222........A.CG.
HPV33|Z066|B	...TCT.C..TT.	....TGT	..A..T..........G..CCAG..C...G.	..T..G.GG.....TG..CGTCT	..TG..	.T...	---	...TCA..A..TTT..G.T.C....C.C	.AG.GA....TA...T......T......GG	.GC.....C.CC.A...C.A.T.T.A.AG-A.222........A.CG.
HPV33|Qv23819|B	...TCT.C..TT.	A.C.TGT	................G..CC.G..C...G.	.CT.AG.GG..C..TG...GTCT	..TG..	.T...	TT-	...TCA..A.TTTTG.G.T.C....C.C	.AG.GA.G..TA...T.G....T......GG	.GC.....C.CC.A..GC.A.T.T.A.AG-A.222...C....A.CG.
HPV33|BF131|B	T..TCT.C..TT.	..C.TGT	.G.........G....G..CC.G..C...G.	..T..G.GG...C.TG...GTCT	C.TG..	.T...	---	TT.TCA..A..TTTG.G.T.C....C.C	.AG.GA....TA...T......T......GG	.GC.....C.CCCA...C.A.T.T.A.AG-A.222.....A..A.CG.
HPV33|Qv34189|B	..ATCT.C..TT.	..C.TGT	................G..CC.G..C...G.	..T..GCGG...C.TG...G.CT	C.TG..	CT...	---	...TCA.GA..TTTG.G.T.C....C.C	.AG.GA....TAC..T......T......GG	.GC..C..C.CC.A...C.A.T.T.A.AG-..222........AACG.

	E6	E7	E1	E2	E4	E5	L2	L1
	1 1		1111 2 3 4 5 556	1111 2 222 23 3			1 1 1 33 33	11 2 22 3 3 4 4
	33 78891 3	14 99	466 2356 1 3 6 6 692	4788 5667 1 334 90 2	2 44	457	127 70 3 6 45 67	5 8 33 1 66 5 8 7 9
	56 33634 8	25 37	4 138 9697 1 7 4 7 964	5 3239 1016 7 071 62 6	5 58	050	6334 73 1 1 10 02	6 7 35 9 68 7 5 2 5
HPV33|Ref|A1	KP IVNKE A	VA TQ	P DTA ETLD S T S S WAH	S LVST KIHA D PAT YS V	I PL	WLH	SSAI DS V P ED DN	T P GK D TG E E G K
HPV33|Qv32494|A1	.. ..	.. ..	. ... G	. ...	. ..	R..	.... .P	. . ..
HPV33|Qv22751|A1	.. ..	.. ..	. ...	. ...	. ..	...	.... K. ..	. . .. E .. . . . .
HPV33|Qv35834|A1	.. ....V .	.. ..	. ...	. ...	. ..	.V.	....	. . ..
HPV33|BF266|A1	.. ..	.. ..	. ...	. ...	. ..	...	....	. . ..
HPV33|RW702|A1	.. ..	.. ..	. ...	. ...	. ..	...	.... N.	. . ..
HPV33|Z084|A1	.. ..	.. ..	. ... K...	. ...	. ..	...	.... N.	. . ..
HPV33|RW926|A1	.. ..	.. ..	. ...	. ...P .. .	. .F	...	.... N.	. . ..
HPV33|Qv18347|A1	N.	.. .L	. ...	. ...	. ..	...	.... N. . . .N ..	. . .R . KE . . . .
HPV33|Qv06895|A1	N.	.. .L	. .A. .A.. . . . . C..	. ...	. ..	...	..V. N. . . .N ..	. . .R . KE . . . .
HPV33|AS099|A1	N. ...N. .	.E ..	. ...	. ...Y.	. ..	...	.F.. N.	. . .R . KE . . . .
HPV33|Qv34060|A2	N. ..H.. .	.. .L	. ... .V.	. F... .V.V . ..P .C S	. .F	..Y	.... N. . . .H NT	N . S. . K. . D . .
HPV33|INCC0137|A2	N. ..H.. .	.. .L	. ... .V.	. F... .V.V . ..P .C S	. .F	..Y	.... N. . . .H NT	N . S. . K. . . . .
HPV33|INJP06456|A2	N. ..H.. .	.. .L	. ... .V.	. F... .V.V . ..P .C S	. .F	..Y	.... N. . . .H NT	N . S. . K. . . . .
HPV33|Qv32431|A2	N. ..H.. .	.. .L	S Y.. .... . A . . .V.	L FA.. .V.. . ..P .C S	. .F	...	.... N. . . .H NT	N S S. . K. . . D .
HPV33|RW136|B	.. LL.N. V	I. SL	. ... ..I. . . . P ..D	. F..A EV.. . SG. .C S	. A.	...	FF.. N. I . .H .T	N . S. . K. . . . R
HPV33|IN241890|B	.. LL.N. V	.. SL	. ..V .... P ..D	. F..A EV.. . SG. HC S	. A.	...	.... N. I . .H .T	N . S. . K. . . . R
HPV33|Z066|B	.. LL.N. V	.. SL	. ..V .... . . N P ..D	. F..A EV.. . SG. HC S	. A.	...	.... N. I . .H .T	N . S. . K. . . . R
HPV33|Qv23819|B	.. LL.N. V	I. SL	. ... P ..D	. F.NA EV.. . SG. .C S	. A.	...	.... N. I S .H .T	N . S. . K. . . . R
HPV33|BF131|B	.. LL.N. V	.. SL	. ...E . . . P ..D	. F..A EV.. A SG. .C S	L A.	...	FF.. N. I . .H .T	N . S. . K. . . . R
HPV33|Qv34189|B	.T LL.N. V	.. SL	. ... P ..D	. F..A EV.. A SG. .C A	L A.	...	.... N. I . .H .T	N . S. . N. . . . R

Note:
1 an insertion of 8 bp (ATACCCTA) between sites nt. 7533 and 7534 within the URR region;
2 a deletion of 79 bp between sites nt. 7583 and 7661 within the URR region.

B

	E6	E7	E1	E2	E4	E5	NCR2	L2	L1	URR
			111111122222	222333333333333333	333333	3334	44444	444444444444445555555555	555555566666666666666667	77777777777777777777777
	1111122333355	667	89334668801237	799111123344446666	334444	8890	11111	122333566668990112222335	567889911223333445666770	01112233334445556666778 111
	2233729245704	274	98886051758432	268344622734674799	273467	2543	01223	916048925691899040467158	893891344050259370344340	958969138911823313671571123678999000
	4716995616083	858	48364916225798	870127956711507725	671150	1685	97572	754454547360942162559344	495842949953968100226737	460816532418015846249835636462125348
HPV35|REF|A1	ATCTCTAATGGAG	TTG	AGTTAACGTTGTAT	TCCACTGCGGCTACGAAG	GGCTAC	ATTC	TTTTC	CGAGTAAAAGCTAGGTACTCGCTC	CCGGGAGGAAGCAGTTTCTTGAAA	ACGCAGAGGA-AT-TTTCTCTACCAGCGAGAGA-TG
HPV35|Qv19083|A1	..A...T......	...	....	.......A..........	......	....	....T	....	.............A..........	..........-..-...................-..
HPV35|QV24067|A1	..A...TG....T	...	....	.......A..........	......	....	.....	....................A...	.............A..........	..........-G.-...................-..
HPV35|Qv19086|A1	..A...TG...G.	...	....	.......A..........	......	C...	.....	.............A....C.....	.............A..........	..........-G.-.....T......T......-..
HPV35|Qv24860|A1	G.A...T......	...	....	..A....A.......C..	......	....	.....	.................A......	......A......A...A......	..........-..-C..................-..
HPV35|Z141|A1	..A...T......	...	...A........G.	..A....A.......C..	......	....	.....	...............C........	......AC...T.A...A......	....C....C-..-C..................-..
HPV35|Z049|A1	.CAC..T.C....	...	...A.........G	G.A....A.A..G..C..	.A..G.	....	.G..T	...A.G.........C........	....A.A......A...A.A....	...2....A.3.--C.C...GG...........-..
HPV35|Rw632|A1	.CAC..T.C....	...	...A.........G	G.A....A....G.AC..	....G.	....	.....	...A.G.........C........	....A.A......A...A.A....	..T.......3.--C......G...........-..
HPV35|QV28721|A1	.CAC..T.C...T	...	G..A.........G	G.A....A....G..C..	....G.	....	.G.GT	...A.G.........C........	....A.A......A...A.A....	...2......3.--C.....GG...........-..
HPV35|QV24366|A1	.CACT.T.C...T	...	...A..TA.....G	G.A....A....G..C..	....G.	....	.....	.A.A.G..G......C........	....A.A......A...A.A....	..........4.--C......G....T......-..
HPV35|IN221713|A1	.CAC..T.C.C..	...	...A....C....G	G.A....AA...G..C..	A...G.	..C.	.....	...A.G.........C........	....A.A.....CAA..A.A....	..........4.--C......G...........-..
HPV35|BF266|A1	.CAC..T.C....	...	...A.........G	G.A....AA...G..C..	A...G.	....	.....	...ACG.........C........	....A.A......A...A.A....	.....A....4.--C......G...........-..
HPV35|Rw807|A1	.CAC..T.C....	...	...A.........G	G.A....A....G..C..	....G.	....	.....	...A.G......C..C........	....A.A......A...A.A.T..	..T.......4.--C..7...G...........-..
HPV35|Rw862|A1	.CAC..T.C....	...	...A.........G	G.A....A....G..C..	....G.	....	.....	...A.G......C..C........	....A.A......A...A.A.T..	..T.......4.--C..7...G...........-..
HPV35|Rw656|A2	..A...T......	.C.	..GA.G.....C..	..A..C.A....GT.CC.	....GT	....	.C.C.	.......T.ATC..ACC....A.T	T.....A......A...A..C..G	..........-..-CC.......A.A..C....-..
HPV35|BF313|A2	..A...T..T...	CCA	...A.G...C....	..A..CAA....G..C..	....G.	.G.T	...C.	T......T.A.C..ACC....A.T	T.A...A..G...A...A.....G	G.........-..-C........A.A.......8..
HPV35|BF223|A2	..A...T......	.C.	...A.G.......G	G.A..C.AA..CG..C..	A..CG.	....	1.GC.	T.G....T.A.C..ACC....A.T	T.A...A......A..CA....GG	..........-..-C......G.A.A.......-..
HPV35|IN272089|A2	..A...T......	.C.	...A.G.......G	G.A..C.AA..CG..C..	A..CG.	....	...C.	T.G....T.A.C..ACC....A.T	T.A...A......A..CA....GG	..........-..-C......G.A-A.......-..
HPV35|BF314|A2	..A...T......	.CA	...ACG....C...	..A.GC.A..TCG..C..	..TCG.	....	...C.	T......T.A.C..ACC....A.T	T.AA..A......A...A.....G	......GA..-..-C........A.A....G..-..
HPV35|BF025|A2	..A...T......	.CA	...ACG....C...	.AA.GC.AA.TCG..C..	A.TCG.	.G.T	...C.	T......T.A.C..ACC....A.T	T.AA..A......A...A.....G	......GA..-..-C........A.A....G..-..
HPV35|Rw128|A2	..A..CT......	CCA	...A.G........	..AT.C.A...CG..C..	...CG.	....	...CT	.......T.A.C..ACC....A.T	TTA...A.T..T.A.C.AC....G	.A........-..-C...G...TA.A.......-..
HPV35|Z147|A2	..A...T......	.C.	...A.G........	..AT.C.A...CG..C..	...CG.	....	...CT	......CT.A.C..ACC....AGT	TTA...A.T..T.A.C.A.....G	.A........-..6C........A.A.......-..
HPV35|QV29782|A2	..A...T.....T	CC.	...A.G........	..AT.C.A...CG..C.A	...CG.	....	...C.	.......T.A.C..ACC..T.A.T	TTA...A.T.A..A...A.....G	.A........5..-C........A.A.A.A.AT-GA
HPV35|QV31639|A2	..A...T.....T	CC.	.A.A.G........	..AT.C.A...CG..C..	...CG.	....	...C.	.......T.A.C..ACC....A.T	TTA..CA.T....A...A.....G	.A........5..-C........A.A.A.A.AT-GA

	E6					E7		E1									E2																E4		E5		L2									L1			
	24	73	78	83	145	23	63	41	172	173	199	248	317	440	491	621	621	85	89	140	143	145	171	205	222	240	243	251	253	312	327	328	50	58	3	15	2	32	260	262	331	352	357	368	382	182	219	348	349
HPV35|REF|A1	H	I	W	V	R	Y	E	D	H	D	E	I	E	V	K	F	F	T	S	I	D	I	T	E	E	H	L	Y	P	A	N	D	F	T	D	L	R	V	N	D	T	S	D	T	L	A	D	S	S
HPV35|Qv19083|A1	.	.	.	.	.	.	.	.	.	.	.	.	.	.	.	.	.	.	.	.	.	.	N	.	.	.	.	.	.	.	.	.	.	.	.	.	.	.	.	.	.	.	.	.	.	.	.	.	.
HPV35|QV24067|A1	.	V	.	.	I	.	.	.	.	.	.	.	.	.	.	.	.	.	.	.	.	.	N	.	.	.	.	.	.	.	.	.	.	.	.	.	.	.	.	.	.	.	N	.	.	.	.	.	.
HPV35|Qv19086|A1	.	V	.	.	.	.	.	.	.	.	.	.	.	.	.	.	.	.	.	.	.	.	N	.	.	.	.	.	.	.	.	.	.	.	A	.	.	.	.	N	.	.	.	.	.	.	.	.	.
HPV35|Qv24860|A1	.	.	.	.	.	.	.	.	.	.	.	.	.	.	.	.	.	.	R	.	.	.	N	.	.	.	.	.	.	.	.	.	.	.	.	.	.	.	.	.	N	.	.	.	.	.	.	.	.
HPV35|Z141|A1	.	.	.	.	.	.	.	.	.	E	.	.	.	.	R	.	.	.	R	.	.	.	N	.	.	.	.	.	.	.	.	.	.	.	.	.	.	.	.	.	.	.	.	.	.	P	.	.	.
HPV35|Z049|A1	.	.	R	.	.	.	.	.	.	E	.	.	.	.	.	V	V	.	R	.	.	.	N	.	K	.	.	C	.	.	.	.	.	A	.	.	.	I	.	.	.	.	.	.	.	.	.	T	.
HPV35|Rw632|A1	.	.	R	.	.	.	.	.	.	E	.	.	.	.	.	V	V	.	R	.	.	.	N	.	.	.	.	C	.	T	.	.	.	A	.	.	.	I	.	.	.	.	.	.	.	.	.	T	.
HPV35|QV28721|A1	.	.	R	.	I	.	.	.	.	E	.	.	.	.	.	V	V	.	R	.	.	.	N	.	.	.	.	C	.	.	.	.	.	A	.	.	.	I	.	.	.	.	.	.	.	.	.	T	.
HPV35|QV24366|A1	Y	.	R	.	I	.	.	.	.	E	.	.	K	.	.	V	V	.	R	.	.	.	N	.	.	.	.	C	.	.	.	.	.	A	.	.	Q	I	.	.	.	.	.	.	.	.	.	T	.
HPV35|IN221713|A1	.	.	R	.	.	.	.	.	.	E	.	.	.	.	.	V	V	.	R	.	.	.	N	K	.	.	.	C	.	.	.	.	.	A	.	.	.	I	.	.	.	.	.	.	.	.	.	T	.
HPV35|BF266|A1	.	.	R	.	.	.	.	.	.	E	.	.	.	.	.	V	V	.	R	.	.	.	N	K	.	.	.	C	.	.	.	.	.	A	.	.	.	I	.	.	.	.	.	.	.	.	.	T	.
HPV35|Rw807|A1	.	.	R	.	.	.	.	.	.	E	.	.	.	.	.	V	V	.	R	.	.	.	N	.	.	.	.	C	.	.	.	.	.	A	.	.	.	I	T	.	.	.	.	.	.	.	.	T	.
HPV35|Rw862|A1	.	.	R	.	.	.	.	.	.	E	.	.	.	.	.	V	V	.	R	.	.	.	N	.	.	.	.	C	.	.	.	.	.	A	.	.	.	I	T	.	.	.	.	.	.	.	.	T	.
HPV35|Rw656|A2	.	.	.	.	.	.	.	.	Q	E	.	V	.	.	.	.	.	.	R	.	.	T	N	.	.	.	.	C	S	.	H	.	.	A	.	.	.	.	.	.	.	.	.	N	.	.	.	.	T
HPV35|BF313|A2	.	.	.	L	.	H	K	.	.	E	.	V	.	.	.	.	.	.	R	.	.	T	N	.	.	.	.	C	.	.	.	.	.	A	.	V	.	.	.	.	.	.	.	N	.	.	.	.	.
HPV35|BF223|A2	.	.	.	.	.	.	.	.	.	E	.	V	.	.	.	V	V	.	R	.	.	T	N	K	.	.	P	C	.	.	.	.	L	A	.	.	.	.	.	.	.	.	.	N	.	.	.	.	.
HPV35|IN272089|A2	.	.	.	.	.	.	.	.	.	E	.	V	.	.	.	V	V	.	R	.	.	T	N	K	.	.	P	C	.	.	.	.	L	A	.	.	.	.	.	.	.	.	.	N	.	.	.	.	.
HPV35|BF314|A2	.	.	.	.	.	.	K	.	.	E	D	V	.	L	.	.	.	.	R	.	E	T	N	.	.	Y	P	C	.	.	.	.	L	A	.	.	.	.	.	.	.	.	.	N	.	.	.	.	.
HPV35|BF025|A2	.	.	.	.	.	.	K	.	.	E	D	V	.	L	.	.	.	K	R	.	E	T	N	K	.	Y	P	C	.	.	.	.	L	A	.	V	.	.	.	.	.	.	.	N	.	.	.	.	.
HPV35|Rw128|A2	.	.	.	.	.	H	K	.	.	E	.	V	.	.	.	.	.	.	R	L	.	T	N	.	.	.	P	C	.	.	.	.	L	A	.	.	.	.	.	.	.	.	.	N	.	.	.	.	.
HPV35|Z147|A2	.	.	.	.	.	.	.	.	.	E	.	V	.	.	.	.	.	.	R	L	.	T	N	.	.	.	P	C	.	.	.	.	L	A	.	.	.	.	.	.	.	.	.	N	V	.	.	.	.
HPV35|QV29782|A2	.	.	.	.	I	H	.	.	.	E	.	V	.	.	.	.	.	.	R	L	.	T	N	.	.	.	P	C	.	.	.	N	L	A	.	.	.	.	.	.	.	L	.	N	.	.	N	.	.
HPV35|QV31639|A2	.	.	.	.	I	H	.	N	.	E	.	V	.	.	.	.	.	.	R	L	.	T	N	.	.	.	P	C	.	.	.	.	L	A	.	.	.	.	.	.	.	.	.	N	.	.	.	.	.

Note:
1 deletion of 3 bp between sites nt. 4109 and 4111 within the NCR2 region;
2 deletion of 11 bp between sites 7198 and 7208 within the URR region;
3 insertion of 16 bp (TTTTACCTCCATTTTG) between sites 7411 and 7412 within the URR region;
4 insertion of 16 bp (TTCTACCTCCATTTTG) between sites 7411 and 7412 within the URR region;
5 insertion of 29 bp (TGTGTTAGTGTCATTCTACCTCCATTTTG) between sites 7411 and 7412 within the URR region;
6 insertion of 20 bp (CACAACACTTAATCCTTGTG) between sites 7521 and 7522 within the URR region;
7 deletion of 9 bp between sites 7636 and 7644 within the URR region;
8 insertion of 2 bp (GG) between sites 103 and 104 within the URR region;

C

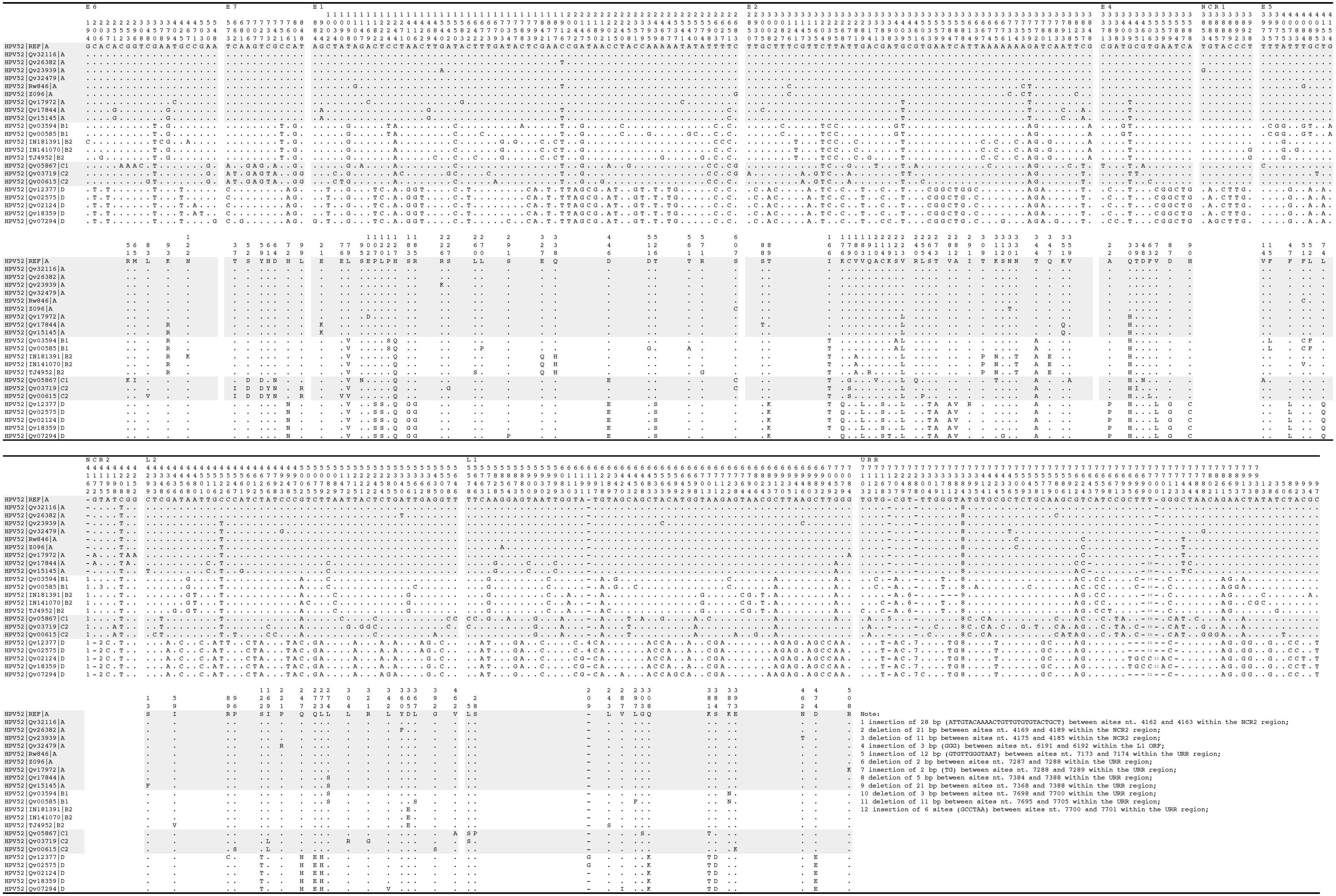
E6
E7
E1
E2
E4
NCR1
E5
NCR2
L2
L1
URR
Note:
1 insertion of 28 bp (ATTGTACAAAACTGTTGTGTGTACTGCT) between sites nt. 4162 and 4163 within the NCR2 region;
2 deletion of 21 bp between sites nt. 4169 and 4189 within the NCR2 region;
3 deletion of 11 bp between sites nt. 4175 and 4185 within the NCR2 region;
4 insertion of 3 bp (GGG) between sites nt. 6191 and 6192 within the L1 ORF;
5 insertion of 12 bp (GTGTTGGGTAAT) between sites nt. 7173 and 7174 within the URR region;
6 deletion of 2 bp between sites nt. 7287 and 7288 within the URR region;
7 insertion of 2 bp (TG) between sites nt. 7288 and 7289 within the URR region;
8 deletion of 5 bp between sites nt. 7384 and 7388 within the URR region;
9 deletion of 21 bp between sites nt. 7368 and 7388 within the URR region;
10 deletion of 3 bp between sites nt. 7698 and 7700 within the URR region;
11 deletion of 11 bp between sites nt. 7695 and 7705 within the URR region;
12 insertion of 6 sites (GCCTAA) between sites nt. 7700 and 7701 within the URR region;

D

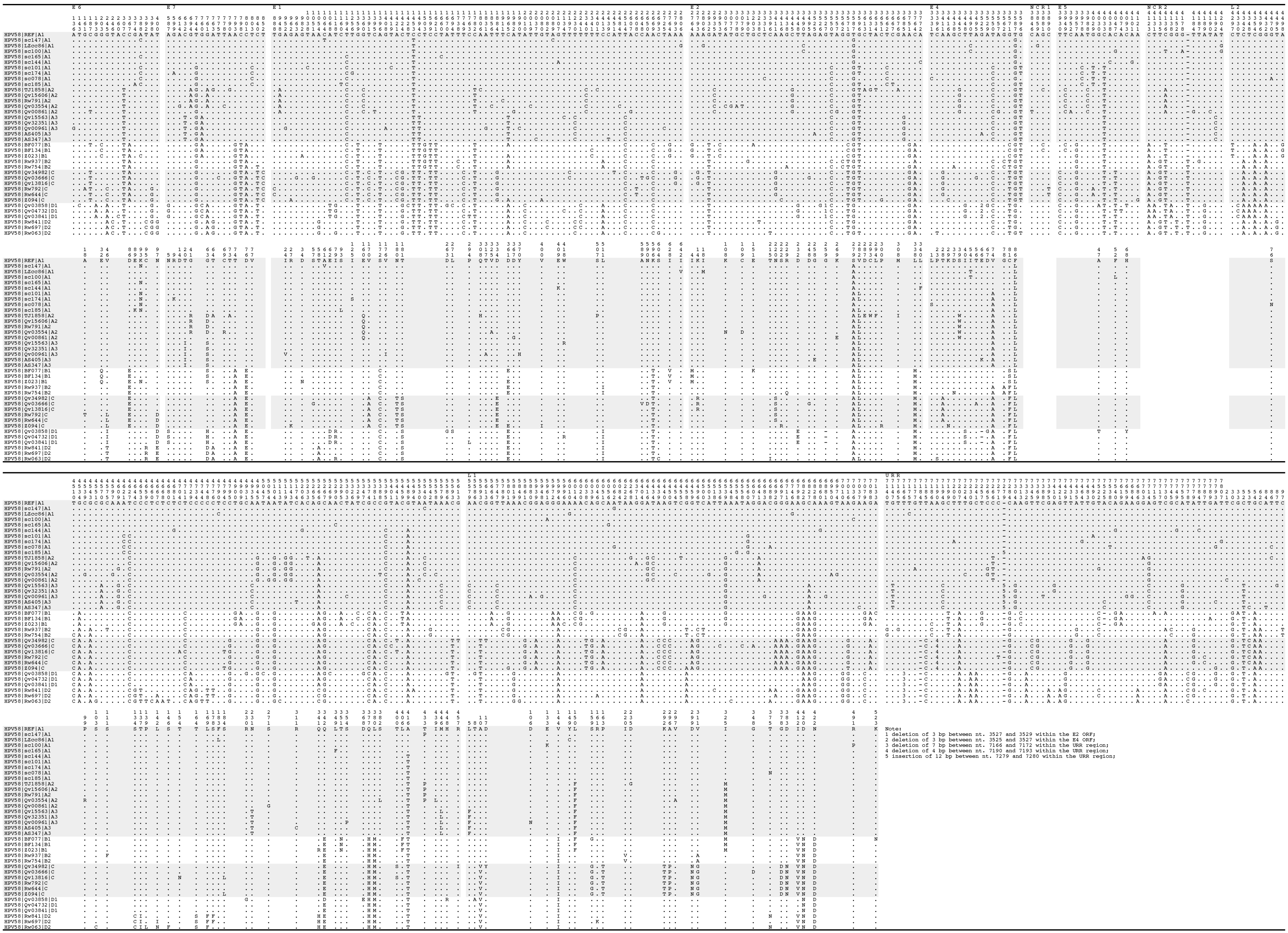
Note:
1 deletion of 3 bp between nt. 3527 and 3529 within the E2 ORF;
2 deletion of 3 bp between nt. 3525 and 3527 within the E4 ORF;
3 deletion of 7 bp between nt. 7166 and 7172 within the URR region;
4 deletion of 4 bp between nt. 7190 and 7193 within the URR region;
5 insertion of 12 bp between nt. 7279 and 7280 within the URR region;

E

	E6	E7	E1	E2	E4	E5	NCR2	L2	L1	URR
			11111111111111122222222222222	2223333333333333333	333333	334	444444	444444444455555555	555556666666666666666777	77777777777777777
	11222344	7777778	800111345556677900112233333467	7880122244444466777	444444	991	111122	234456688912222344	677790111144456666779001	11133344444556667
	03155705	1157794	936056171590668300991100134823	3679423911135816028	111358	020	358823	664868959820123645	604817569903760127583271	66701533899069999344
	75724158	2425894	800945973243580326783829291833	3590389013460545418	135605	923	932445	297047442049552132	622727311877493192282567	12983468539953780956
HPV67|REF|A1	TCGAAACT	TCGTCAC	CTTCGTTGTTCATGTCATTTATCTCGCGCG	GCGGGACTAA-GGATTTCC	AA-GGA	ACA	GTGCT-	CTTAATCCCGATGCTAAA	TATAAAAGGGAACAATAGCATGTA	TGG-GAGACC-CGTAACATG
HPV67|QV25560|A1	.TC..CA.	A......	......G.........T....CT......T	TA........-AAT....A	..-AAT	...	A....A	........T.G.......	.TC..G..A..........C..GG	...GA.C...3...G.....
HPV67|QV31430|A1	.T...CA.	A......	......G.........T....CT......T	TA........-........A	..-...	...	A....A	.....G..T.G.T.....	.TC..GG.............C.GG	...GA.C...3...G.....
HPV67|QV22701|A2	.T....A.	.TC.G.T	...T.C................T.....TT	TA....TC..-.....AGA	..-...	CT.	A..T.A	A..T....T......C..	.TC.GG...A...GC.......G.	.A.GA.....-...G..GG.
HPV67|QV33023|A2	.T....A.	.T..G..	...T.C................T.....TT	TA....TC..-.....AGA	..-...	.T.	A..T.A	...T....T......C..	.TC..G...A...GC.......G.	.A.GA...T.-...G..GG.
HPV67|QV24827|B	CT.CC.AC	..CC...	TCG.A..TCCTGC.CA.CCCC.TCATTT.T	TAAAACT.GG1...C..GA	GG2...	..C	A.A.CA	.CA.G.GATA....C.TG	ATCT.G.C.......CGC...AGG	..AGAG.G.A-TACGG...A
HPV67|QV25738|B	CT.CC.AC	..CC.G.	TC..A..TC.TGC..A.C.CC.TCATTT.T	TAAAAC...G1...CC.GA	.G2...	..C	AGA..A	.CA.G.GAT....TC.T.	ATCT.G....GTT...GCT...G.	...GAG.G.A-TACGGT..A
HPV67|QV30703|B	CT.CC.AC	..CC.G.	TC..A..TC.TGCA.A.CCCC.TCATTT.T	TAAAACT..G1...CC.GA	.G2...	..C	AGA..A	.CA.G.GAT..C..C.T.	ATCT.G....GTT...GCT...G.	C..GAG.G.A-TACGGT..A

	E6	E7	E1	E2	E4	E5	NCR2	L2	L1
	39 51 90	50 51 64 72	62 94 149 264 353 447 480 489 620	5 49 124 170 231 231 241 246 330 336	39 39 49 65	4 8		9 153 206 219 248 296 326 330 332 357 375	87 209 221 224 359 382
HPV67|REF|A1	R K E	D T E H	D A C V D K Q A C	A L D K K - R D H A	N - D Y	I A		R Q T P R K Q P V Y	T S M V I A
HPV67|QV25560|A1	T . D	E . . .	. . G . . . . . F	S M . . . - Q N . .	. - N F	. .		. . . S . R	. . I . . .
HPV67|QV31430|A1	. . D	E . . .	. . G . . . . . F	S M . . . -	. - . .	. .		. . . S . R H . . .	. G . . . .
HPV67|QV22701|A2	. . .	. I Q Q	. . . . F	S M . . . - . . Q G	. - . .	L V		S . . S . . . . . S	. . . I L .
HPV67|QV33023|A2	. . .	. I . Q	. . . . F	S M . . . - . . Q G	. - . .	. V		. . . S . . . . . S	. . . I L .
HPV67|QV24827|B	. H .	. . Q .	E T . A E Q K S F	S M N Q E 4 . . . G	S 5 . .	. .		. E N S K . . . A .	S . . . . P
HPV67|QV25738|B	. H .	. . Q .	. T . A E Q K S F	S M N Q . 4 . . . G	S 5 . .	. .		. E N S . . . S A .	S . . . . P
HPV67|QV30703|B	. H .	. . Q .	. T . A E Q K S F	S M N Q . 4 . . . G	S 5 . .	. .		. E N S . . . . A .	S . . . . P

Note:
1 insertion of 6 bp (CATAAA) between sites nt. 3414 and 3415 within the E2 ORF;
2 insertion of 6 bp (ATAAAC) between sites nt. 3415 and 3416 within the E4 ORF;
3 insertion of 16 bp (TCTTGCCAACATTGTT) between sites nt. 7499 and 7500 within the URR region;
4 insertion of 2 amino acids (HK) between sites aa. 231 and 232 within the E2 ORF;
5 insertion of 2 amino acids (IN) between sites aa. 39 and 40 within the E4 ORF.

F
