# Supplementary material for: Evolution and Taxonomic Classification of Human Papillomavirus 16 (HPV16)-Related Variant Genomes: HPV31, HPV33, HPV35, HPV52, HPV58 and HPV67
Source: PLoS One. 2011 May 27;6(5):e20183. doi: 10.1371/journal.pone.0020183 (PMC3103539; doi:10.1371/journal.pone.0020183)
Supplement: Table S1 — List of HPV genomes by type, isolate name, geographic origin of sample, lineage designation, length of complete genome and NCBI #. (PDF) [file pone.0020183.s005.pdf]

Table S1. List of HPV genomes by type, isolate name, geographic origin of sample, lineage designation, length of complete genome and NCBI #.

HPV Type	Isolate ID	Origin	Lineage	Sub-lineage	Length	NCBI #
HPV31	HPV31REF	-	A		7912	J04353*
HPV31	Z014	Zambia	A		7908	HQ537666
HPV31	QV18335	Costa Rica	A		7920	HQ537667
HPV31	IN453545	Thailand	A		7914	HQ537668
HPV31	QV00071	Costa Rica	A		7908	HQ537669
HPV31	QV02784	Costa Rica	A		7908	HQ537670
HPV31	QV00215	Costa Rica	A		7908	HQ537671
HPV31	QV02689	Costa Rica	A		7902	HQ537672
HPV31	QV00168	Costa Rica	A		7902	HQ537673
HPV31	QV14117	Costa Rica	A		7942	HQ537674
HPV31	IN221709	Thailand	A		7945	HQ537675
HPV31	QV03876	Costa Rica	B		7898	HQ537676
HPV31	QV06582	Costa Rica	B		7898	HQ537677
HPV31	RW687	Rwanda	B		7895	HQ537678
HPV31	IN231831	Thailand	B		7892	HQ537679
HPV31	QV17700	Costa Rica	B		7886	HQ537680
HPV31	QV05335	Costa Rica	B		7892	HQ537681
HPV31	QV03136	Costa Rica	C		7878	HQ537682
HPV31	BF363	Burkina Faso	C		7878	HQ537683
HPV31	QV14043	Costa Rica	C		7878	HQ537684
HPV31	QV00693	Costa Rica	C		7878	HQ537685
HPV31	QV13734	Costa Rica	C		7878	HQ537686
HPV31	QV12357	Costa Rica	C		7878	HQ537687
HPV33	HPV33REF	-	A	A1	7909	M12732*
HPV33	QV32494	Costa Rica	A	A1	7910	HQ537688
HPV33	QV22751	Costa Rica	A	A1	7912	HQ537689
HPV33	QV35834	Costa Rica	A	A1	7912	HQ537690
HPV33	BF266	Burkina Faso	A	A1	7909	HQ537691
HPV33	RW702	Rwanda	A	A1	7909	HQ537692
HPV33	Z84	Zambia	A	A1	7909	HQ537693
HPV33	RW926	Rwanda	A	A1	7909	HQ537694
HPV33	QV18347	Costa Rica	A	A1	7911	HQ537695
HPV33	QV06895	Costa Rica	A	A1	7912	HQ537696
HPV33	AS099	China Taiwan	A	A1	7908	HQ537697
HPV33	QV34060	Costa Rica	A	A2	7831	HQ537698
HPV33	INCC0137	Thailand	A	A2	7830	HQ537699
HPV33	INJP06456	Thailand	A	A2	7830	HQ537700
HPV33	QV32431	Costa Rica	A	A2	7833	HQ537701
HPV33	RW136	Rwanda	B		7838	HQ537702
HPV33	IN241890	Thailand	B		7830	HQ537703
HPV33	Z066	Zambia	B		7830	HQ537704
HPV33	QV23819	Costa Rica	B		7832	HQ537705
HPV33	BF131	Burkina Faso	B		7830	HQ537706
HPV33	QV34189	Costa Rica	B		7830	HQ537707
HPV35	HPV35REF	-	A	A1	7879	M74117*
HPV35	QV19083	Costa Rica	A	A1	7879	HQ537708
HPV35	QV24067	Costa Rica	A	A1	7879	HQ537709
HPV35	QV19086	Costa Rica	A	A1	7879	HQ537710
HPV35	QV24860	Costa Rica	A	A1	7879	HQ537711
HPV35	Z141	Zambia	A	A1	7879	HQ537712
HPV35	Z049	Zambia	A	A1	7883	HQ537713
HPV35	RW632	Rwanda	A	A1	7894	HQ537714
HPV35	QV28721	Costa Rica	A	A1	7883	HQ537715
HPV35	QV24366	Costa Rica	A	A1	7894	HQ537716

HPV35	IN221713	Thailand	A	A1	7894	HQ537717
HPV35	BF266	Burkina Faso	A	A1	7894	HQ537718
HPV35	RW807	Rwanda	A	A1	7885	HQ537719
HPV35	RW862	Rwanda	A	A1	7885	HQ537720
HPV35	RW656	Rwanda	A	A2	7879	HQ537721
HPV35	BF313	Burkina Faso	A	A2	7881	HQ537722
HPV35	BF223	Burkina Faso	A	A2	7876	HQ537723
HPV35	IN272089	Thailand	A	A2	7878	HQ537724
HPV35	BF314	Burkina Faso	A	A2	7879	HQ537725
HPV35	BF025	Burkina Faso	A	A2	7879	HQ537726
HPV35	RW128	Rwanda	A	A2	7879	HQ537727
HPV35	Z147	Zambia	A	A2	7899	HQ537728
HPV35	QV29782	Costa Rica	A	A2	7908	HQ537729
HPV35	QV31639	Costa Rica	A	A2	7908	HQ537730
HPV52	HPV52REF	-	A		7942	X74481*
HPV52	QV32116	Costa Rica	A		7937	HQ537731
HPV52	QV26382	Costa Rica	A		7937	HQ537732
HPV52	QV23939	Costa Rica	A		7937	HQ537733
HPV52	QV32479	Costa Rica	A		7937	HQ537734
HPV52	RW846	Rwanda	A		7937	HQ537735
HPV52	Z096	Zambia	A		7937	HQ537736
HPV52	QV17972	Costa Rica	A		7937	HQ537737
HPV52	QV17844	Costa Rica	A		7933	HQ537738
HPV52	QV15145	Costa Rica	A		7933	HQ537739
HPV52	QV03594	Costa Rica	B	B1	7962	HQ537740
HPV52	QV00585	Costa Rica	B	B1	7951	HQ537741
HPV52	IN181391	Thailand	B	B2	7944	HQ537742
HPV52	IN141070	Thailand	B	B2	7960	HQ537743
HPV52	TJ49	China Tianjing	B	B2	7960	GQ472848*
HPV52	QV05867	Costa Rica	C	C1	7974	HQ537744
HPV52	QV03719	Costa Rica	C	C2	7962	HQ537745
HPV52	QV00615	Costa Rica	C	C2	7962	HQ537746
HPV52	QV12377	Costa Rica	D		7937	HQ537747
HPV52	QV02575	Costa Rica	D		7937	HQ537748
HPV52	QV02124	Costa Rica	D		7951	HQ537749
HPV52	QV18359	Costa Rica	D		7951	HQ537750
HPV52	QV07294	Costa Rica	D		7934	HQ537751
HPV58	HPV58REF	-	A	A1	7824	D90400*
HPV58	LZCC86	China Gansu	A	A1	7824	EU918765*
HPV58	SC100	China Sichuan	A	A1	7824	FJ385261*
HPV58	SC101	China Sichuan	A	A1	7824	FJ385262*
HPV58	SC144	China Sichuan	A	A1	7824	FJ385264*
HPV58	SC147	China Sichuan	A	A1	7824	FJ385263*
HPV58	SC165	China Sichuan	A	A1	7824	FJ385265*
HPV58	SC174	China Sichuan	A	A1	7824	FJ385266*
HPV58	SC185	China Sichuan	A	A1	7836	FJ385267*
HPV58	SC078	China Sichuan	A	A1	7824	FJ385268*
HPV58	TJ18	China Tianjing	A	A2	7824	GQ47285*
HPV58	QV15606	Costa Rica	A	A2	7824	HQ537752
HPV58	RW791	Rwanda	A	A2	7824	HQ537753
HPV58	QV03554	Costa Rica	A	A2	7824	HQ537754
HPV58	QV00861	Costa Rica	A	A2	7824	HQ537755
HPV58	QV15563	Costa Rica	A	A3	7836	HQ537756
HPV58	QV32351	Costa Rica	A	A3	7836	HQ537757
HPV58	QV00961	Costa Rica	A	A3	7836	HQ537758
HPV58	AS405	China Taiwan	A	A3	7836	HQ537759
HPV58	AS347	China Taiwan	A	A3	7836	HQ537760
HPV58	BF077	Burkina Faso	B	B1	7823	HQ537761

HPV58	BF134	Burkina Faso	B	B1	7823	HQ537762
HPV58	Z023	Zambia	B	B1	7823	HQ537763
HPV58	RW937	Rwanda	B	B2	7825	HQ537764
HPV58	RW754	Rwanda	B	B2	7825	HQ537765
HPV58	QV03858	Costa Rica	D	D1	7814	HQ537766
HPV58	QV04732	Costa Rica	D	D1	7814	HQ537767
HPV58	QV03841	Costa Rica	D	D1	7814	HQ537768
HPV58	RW841	Rwanda	D	D2	7817	HQ537769
HPV58	RW697	Rwanda	D	D2	7817	HQ537770
HPV58	RW063	Rwanda	D	D2	7817	HQ537771
HPV58	QV34982	Costa Rica	C		7820	HQ537772
HPV58	QV03666	Costa Rica	C		7820	HQ537773
HPV58	QV13816	Costa Rica	C		7820	HQ537774
HPV58	RW792	Rwanda	C		7820	HQ537775
HPV58	RW644	Rwanda	C		7820	HQ537776
HPV58	Z094	Zambia	C		7820	HQ537777
HPV67	HPV67REF	-	A	A1	7801	D21208*
HPV67	QV25560	Costa Rica	A	A1	7819	HQ537778
HPV67	QV31430	Costa Rica	A	A1	7819	HQ537779
HPV67	QV22701	Costa Rica	A	A2	7803	HQ537780
HPV67	QV33023	Costa Rica	A	A2	7803	HQ537781
HPV67	QV24827	Costa Rica	B		7809	HQ537782
HPV67	QV25738	Costa Rica	B		7809	HQ537783
HPV67	QV30703	Costa Rica	B		7809	HQ537784

* Sequenced obtained from NCBI/GenBank Database.
