# Supplementary material for: Evolution and Taxonomic Classification of Human Papillomavirus 16 (HPV16)-Related Variant Genomes: HPV31, HPV33, HPV35, HPV52, HPV58 and HPV67
Source: PLoS One. 2011 May 27;6(5):e20183. doi: 10.1371/journal.pone.0020183 (PMC3103539; doi:10.1371/journal.pone.0020183)
Supplement: Table S2 — Nucleotide sequence mean difference (± standard error) of HPV16-related alpha-9 HPV complete genomes. The intra-lineage (e.g., A vs. A) and intra-sublineage (e.g., A1 vs. A1) difference values are highlighted in gray. (PDF) [file pone.0020183.s006.pdf]

Table S2. Nucleotide sequence mean difference (± standard error) of HPV16-related alpha-9 HPV complete genomes. The intra-lineage (e.g., A vs. A) and intra-sublineage (e.g., A1 vs. A1) difference values are highlighted in gray.

**HPV31**	A	B	C
A	0.27±0.03		
B	0.86±0.09	0.27±0.04	
C	1.15±0.11	1.19±0.12	0.42±0.05

**HPV33**	A	B
A	0.39±0.04	
B	0.94±0.08	0.17±0.02

**HPV35**	A
A	0.35±0.04

**HPV52**	A	B	C	D
A	0.19±0.03			
B	0.80±0.08	0.43±0.05		
C	0.96±0.10	1.08±0.09	0.54±0.06	
D	1.75±0.15	1.80±0.15	2.00±0.14	0.13±0.03

**HPV58**	A	B	C	D
A	0.47±0.04			
B	1.12±0.08	0.48±0.05		
C	1.40±0.11	1.05±0.09	0.20±0.03	
D	1.35±0.12	0.93±0.08	0.95±0.10	0.42±0.05

**HPV67**	A	B
A	0.36±0.05	
B	1.00±0.09	0.22±0.04

**HPV33**	A1	A2
A1	0.24±0.03	
A2	0.62±0.07	0.13±0.03

**HPV35**	A1	A2
A1	0.21±0.03	
A2	0.48±0.06	0.24±0.03

**HPV52**	B1	B2	C1	C2
B1	0.13±0.04			
B2	0.59±0.08	0.20±0.04		
C1			n/c	
C2			0.62±0.07	0.39±0.07

**HPV58**	A1	A2	A3	B1	B2	D1	D2
A1	0.24±0.03						
A2	0.60±0.06	0.27±0.03					
A3	0.54±0.07	0.70±0.08	0.14±0.03				
B1				0.16±0.04			
B2				0.71±0.07	0.12±0.04		
D1						0.15±0.03	
D2						0.58±0.07	0.22±0.04

**HPV67**	A1	A2
A1	0.27±0.04	
A2	0.45±0.07	0.08±0.04
